# Transfer of knowledge to diagnose infant abuse and its incidence – a time-series analysis from Sweden

**DOI:** 10.1186/s13012-022-01188-6

**Published:** 2022-02-04

**Authors:** Ulf Högberg

**Affiliations:** 1 Department of Women’s and Children’s Health, Uppsala University, SE-751 85 Uppsala, Sweden; 2 Department of Epidemiology and Global Health, Umeå University, Umeå, Sweden

**Keywords:** Abuse, Implementation science, Infant, Knowledge, Professional practice, Shaken baby syndrome

## Abstract

**Aim:**

To analyse the transfer of knowledge on how to detect physical abuse, especially shaken baby syndrome/abusive head trauma (SBS/AHT), and its association to trends in infant abuse diagnoses (maltreatment and assault).

**Methods:**

Design: retrospective population-based and quasi-experimental. Setting: Sweden 1987–2019. Patients: Children below age 1 year, selected from the National Patient Register (*n* = 1150). Exposures: Literature search for transfer of knowledge by diffusion, dissemination and implementation, and whether supportive or disruptive of the SBS/AHT paradigm. Main outcome measure: Abuse diagnoses (maltreatment or assault). Analyses: Incidence rate, incidence rate ratio (IRR).

**Results:**

The overall incidence rate of abuse was 32.23 per 100,000 during the years 1987–2019. It was rather stable 1987–2000. The SBS diagnosis was introduced in the late 1990s. A comprehensive increase of transfer of knowledge on physical abuse, specifically on SBS/AHT and dangers of shaking, took place from 2002 and onward through diffusion, dissemination and implementation. Maltreatment diagnoses, but not assault diagnosis, increased steeply during 2002–2007, peaking in 2008–2013 [IRR 1.63 (95% confidence interval 1.34–1.98)]. Transfer of disruptive knowledge on SBS/AHT during the period 2014–2019 was associated with a decline in maltreatment diagnoses [IRR 0.84 (95% confidence interval 0.71–0.99)].

**Conclusion:**

An increase in maltreatment diagnoses was associated with transfer of supportive knowledge of the SBS/AHT paradigm, while a decline occurred toward the end of the study period, which might indicate a burgeoning de-implementation process.

**Supplementary Information:**

The online version contains supplementary material available at 10.1186/s13012-022-01188-6.

Contributions to the literature

- Implementation of new diagnostic knowledge into healthcare practice is seldom a straightforward process, but is facilitated if guidance is based on stated objective findings.
- This study shows that a comprehensive national transfer of knowledge on the shaken baby syndrome/abusive head trauma (SBS/AHT) paradigm was associated with a major increase in incidence of infant maltreatment diagnoses, which might encompass hidden cases and/or false positives. When disruptive findings on the diagnostic process in alleged SBS/AHT were introduced, the incidence started to decline.
- Healthcare should have preparedness for changes in practices based on new evidence-based knowledge, until the practices eventually reach obsolescence.

## Background

A high diagnostic precision in the detection of child abuse is of utmost societal importance to keep both false positives and false negatives at a minimum [1], as the consequences can be severe in both cases. The evolving field of knowledge on how to detect infant abuse embraces the developments of modern medicine. Battered-child syndrome was first described by the paediatrician Kempe (1962) as exhibiting external signs of injury, fractures (also metaphyseal and ribs), subdural haemorrhage (SDH) and commonly being associated with parental psychiatric factors. Reference was also made to findings described by the radiologist Caffey in 1940s [2]. The neurosurgeon Guthkelch (1971) hypothesised that repeated acceleration/deceleration caused SDH and retinal haemorrhage (RH) in cases of suspected battered-child syndrome without external signs of trauma [3]. This was described by Caffey (1974) as the whiplash shaken infant syndrome [4]. Later, the term shaken baby syndrome/abusive head trauma (SBS/AHT) was coined, described as part of evidence-based medicine and supported by numerous studies (case reports, case-series, case-control studies and consensus/expert opinions) [5, 6]. Algorithms to predict abuse from radiological or ocular findings [7] were provided, to be implemented into medical and legal practice [6].

Transfer of research findings and innovations into medical practice can be distinguished into phases: diffusion, dissemination, implementation and sustainability until obsolescence [8]. Important implementation drivers promoting uptake of research findings into practice include: capacity building and institutionalisation [9], champions and leadership [8, 10], interdisciplinary approaches [11] and a shared agenda between different stakeholders [8, 12]. However, diagnostic work is seldom a straightforward endeavour of applying evidence; professionals’ understanding, established routines and scientific controversies may influence the uptake of new research findings into medical practice [13].

Disruptive reasoning and findings have challenged the evidence on the SBS/AHT paradigm. The proposed shaking mechanism causing SDH and RH has been questioned [14–20], and differential diagnoses for rib fractures and metaphyseal lesions have been presented [21]. Further, a major bias, circular reasoning, has been identified by two systematic reviews – concluding that there is insufficient scientific evidence to assess the diagnostic accuracy of the triad findings (SDH, RH and encephalopathy) in identifying traumatic shaking [22], and to determine if rib fractures or metaphyseal lesions are caused by abuse [23]. Thus, the state of knowledge on the SBS/AHT paradigm has been, and still is, subject to scientific controversy and can be described as undergoing a paradigm shift [24–27].

The rationale for this study was to advance the understanding of how health services are implementing evolving research findings in the field of child protection. We have previously reported on an association between SBS/AHT criteria and infant maltreatment diagnosis, and a 10-fold increased incidence of maltreatment diagnosis among infants born 1987–2014 [28]. There is a lack of studies exploring the relationship between scientific/medical controversies on evidence, which play out in scientific journals and grey literature, and the practices of the medical professionals who manage the controversial matters in daily medical practice.

The objective was to analyse the transfer of knowledge on how to detect physical abuse, especially SBS/AHT, and its association to trends in infant abuse diagnoses (maltreatment and assault) during the years 1987 to 2019.

## Methods

This was a population-based study with a quasi-experimental retrospective design departing from a literature review.

### Setting

Sweden, which currently has a population of 10 million inhabitants, has a decentralised healthcare system with 21 regions providing healthcare services in health centres, about 70 hospitals and 7 university hospitals, each with a catchment area of 1–2 millions. Care is funded mainly through general taxation. National governance occurs through laws, regulations and guidance from government agencies such as the National Board of Health and Welfare, the Swedish Agency for Health Technology Assessment and Assessment of Social Services (SBU), the Swedish National Council on Medical Ethics, the Forensic Advisory Board of the National Board of Health and Welfare and the National Board of Forensic Medicine. Specialist medical professional associations, such as the Swedish Paediatric Society, Swedish Forensic Society, Swedish Ophthalmological Society and the Swedish Society for Paediatric Radiology, as part of Swedish Society of Radiology, are organised in the Swedish Medical Association and the Swedish Society of Medicine; they can issue care guidelines to their members.

### Child protection from the 1950s to the 1980s

Sweden has long focused on promotion of child health and prevention of childhood morbidity and mortality. From the 1950s, national coverage of well-baby clinics was achieved in collaboration with antenatal clinics, and subsequently with social services. Through the work of the Child Accident Prevention Committee, established in 1954 by Professor Ragnar Berfenstam, a dramatic decline in mortality from home and leisure trauma was achieved from the 1950s and onward. The knowledge on child abuse and neglect, the medical diagnosis battered-child syndrome, and actions directed at prevention and early detection came into focus from the late 1950s through case studies, governmental reports, media attention and petitions to the parliament. In 1979, Sweden became the first country to ban corporal punishment and emotional humiliation. Based on knowledge on battered-child syndrome from Caffey (1946) and Kempe (1962), child abuse was described based mainly on signs of external injuries, multiple fractures and – more seldom – SDH. Most cases were reported in metropolitan areas, an increasing trend was seen, and missed cases of abuse were considered plausible. The incidence was estimated to be substantially lower than in the US and the UK. Actions recommended for prevention and early detection had an ecological framing describing individual, family and societal risk factors, as parental social or psychiatric adversities were commonly found to be associated with abuse. Improved teaching of health professionals and social rehabilitation and treatment support to the parents were also suggested (Additional file 1).

### Data sources

The Swedish National Board of Health and Welfare maintains health registers with national coverage. The National Patient Register (NPR) includes information on in-patient care (= hospitalised patients), including dates of admission and diagnoses, with full national coverage from 1987. From 2001, it also includes information on outpatient specialised care, but has no data from primary care. The Death Cause Register comprises information on cause and date of death. The Medical Birth Register contains data on all children born in Sweden. Statistics Sweden provides population data.

### Cases

Cases were defined based on the diagnoses of abuse and its sub-categories maltreatment (suspected maltreatment or diagnosed maltreatment), associated with SBS/AHT criteria [28], and assault, based on the codes in the International Classification of Diseases (ICD-9 SWE 1987–1996, ICD-10 SWE 1997–2019) (Additional file 2). Age was restricted to under 1 year. Included infants were born between 1987, when the NPR achieved full national coverage, through 2019.

### Definition of knowledge and search terms

The definition of knowledge for this study included both *knowing-that knowledge* (research findings on mechanism knowledge and correlation knowledge) and *know-how knowledge* (practice knowledge, such as recommendations and guidelines) [29], as well as supreme court decisions. Accumulation of knowledge can be seen as a mostly evolutionary process, but is sometimes characterised by discontinuities giving room for new insights into mechanisms, i.e., paradigm shifts [29]. Whether knowledge on the SBS/AHT paradigm was supportive or disruptive was categorised in accordance with Wu et al. [30]. Supportive knowledge was defined as knowledge on SBS/AHT claimed as valid, robust and scientifically based. Disruptive knowledge on SBS/AHT was defined as questioning reasoning or scientific argumenting based on current knowledge, new research findings, such as mechanism knowledge, correlation knowledge and systematic reviews, and judicial assessment of evidence in supreme court decisions.

For this study, knowledge about child abuse in the Swedish setting was searched for using the following search terms: Sweden, child protection, infants, physical abuse, neglect, battered-child syndrome, shaken baby syndrome, abusive head trauma (not child sexual abuse), years 1957–2019 (Additional files 1 & 3). Papers indexed in scientific journals (PubMed) and grey literature were searched for: Journal Article, Newspaper Article, Book, Book Section, Electronic Book Section, Conference Proceedings, Report, Government authorities’ report and conference, Interview, Teaching (Audio-visual material), Professional societies’ guidelines, Healthcare Organisation change and Swedish Supreme Court decision. A data extraction table (Additional file 3) of all the literature found includes authors, title, year, study design – if appropriate, topics addressed, whether the items presented the state of knowledge on abuse, were generally and/or specifically supportive or disruptive of SBS/AHT paradigm, and investigations recommended in case of suspicion of abuse.

### Definition of transfer of knowledge

Transfer of knowledge or innovations was categorised as diffusion, dissemination and implementation in accordance with Greenhalgh et al. [8]:

I. Diffusion (passive spread) – research findings, overviews, comments, textbooks and theses, as such knowledge transfer has no specific recipient.
II. Dissemination (active and planned efforts to persuade target groups to adopt an innovation) – research (systematic reviews), because of it being given greater weight in evidence-based medicine, conference proceedings and teaching material for health professionals, as such knowledge transfer has specific recipients.
III. Implementation (active and planned efforts to mainstream an innovation within an organisation) – documents and reports from Swedish government agencies, changes in professional and healthcare organisations, guidelines from professional and government agencies, and Swedish Supreme Court decisions. The rationale for this categorisation was its possible weight in relation to changing health practices.

The concept of *sustainability* was adopted from Greenhalgh et al. [8]: a time-dependent concept, referring to periods when little new transfer of knowledge occurs, with activities keeping an innovation routine until it reaches obsolescence.

The applied definition of transfer of knowledge did not address the learning process, how the knowledge was received by the recipient or how healthcare providers were affected by the presented information. This could not be addressed using the chosen study design.

### Statistical analysis

The descriptive analysis was based on the incidence rate of abuse and its sub-categories maltreatment and assault per 100,000 infants per year (3-year moving averages).

Intervention points and phases of transfer of knowledge were selected for a time-series analysis to assess any impact in the outcome measure of abuse and its sub-categories maltreatment and assault diagnoses. Poisson regression was applied for this analysis. The dependent variable *(Y)* was the incidence of the sub-categories maltreatment and assault per year obtained in each of the subgroups, described by a set of predictor variables. Predictor variables *(X)* were selected: 1) Change of ICD version from version 9 to version 10 (1997); 2) Extension of the NPR to comprising diagnoses in outpatient specialised healthcare (2001); 3) Timepoints defined by different intensities of diffusion, dissemination and implementation of general knowledge about abuse, and specific knowledge about SBS/AHT with a phase of sustainability until obsolescence (2002–2019); 4) Regional differences in diagnoses of maltreatment and assault (1987–2014); 5) Reported inclusion of neuroimaging in investigations of suspected abuse (1987–2014). Hence, time was treated as an independent covariate. Two separate regression models were fitted for each predictor variable, one to each sub-category, using maximum likelihood estimation in the statistical package GLIM (R version 4.0.3).

## Results

**Fig. 1:**
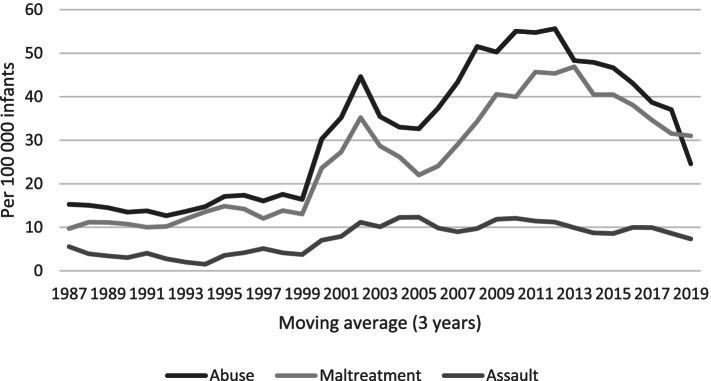
Infants diagnosed with abuse and the subcategories maltreatment and assault during the years 1987 to 2019 in Sweden

The incidences (3-year moving average) were rather stable for both maltreatment and assault diagnoses during the late 1980s and 1990s. Both maltreatment and assault increased around the turn of the century. Assault levelled out, while maltreatment peaked around 2010–2013 (Fig. 1). In all, 1150 cases were found in 1987–2019, 920 had any diagnosis of maltreatment and 230 had a diagnosis of assault; 45 had both. The overall incidence rate was 32.23 per 100,000 infants; the incidence rate of any maltreatment diagnosis was 25.78 and that for any assault diagnosis was 7.43 (Table 1). A three-fold increase was observed in 2001–2019 compared with in 1987–2000 for both maltreatment and assault, 36.15 and 10.33 versus 11.29 and 3.36, respectively (Table 1). By 2001, when outpatient specialised care was included in the NPR, the incidences of maltreatment and assault were 41.46 and 15.28, respectively; in 2000, they were 11.02 and 2.20, respectively.

**Table 1:** Incidence of infant abuse and its subcategories maltreatment and assault during the years 1987 to 2019, divided into the periods 1987–2000 and 2001–2019, cases per 100,000 infants, 95% confidence intervals. Source: National Patient Register covering in-patient care 1987–2000 and, from year 2001, out-patient specialised care as well

	1987–2019	1987–2000	2001–2019
Abuse			
Incidence (95% CI)	32.23 (31.64–32.81)	15.02 (14.39–15.64)	44.51 (43.61–45.41)
Mortality (95% CI)	1.40 (1.36–1.44)	1.88 (1.66–2.10)	1.06 (0.92–1.12)
Maltreatment			
Incidence (95% CI)	25.78 (25.58–26.31)	11.29 (10.75–11.83)	36.15 (35.33–36.97)
Mortality (95% CI)	0.08 (0.07–0.09)	0.07 (0.06–0.08)	0.1 (0.08–0.11)
Assault			
Incidence (95% CI)	7.43 (7.14–7.71)	3.36 (3.07–3.65)	10.33 (9.99–10.77)
Mortality (95% CI)	1.32 (1.20–1.44)	1.81 (1.59–2.03)	0.96 (0.82–1.09)

The overall mortality rate was 1.40 per 100,000 infants; for maltreatment, it was 0.08 (*n* = 3), and for assault, it was 2.16 (*n* = 47). The mortality rate declined by 50% in 2001–2019 compared with in 1987–2000 due to a decline in assault deaths, while maltreatment deaths remained unchanged (Table 1).

**Fig. 2:**
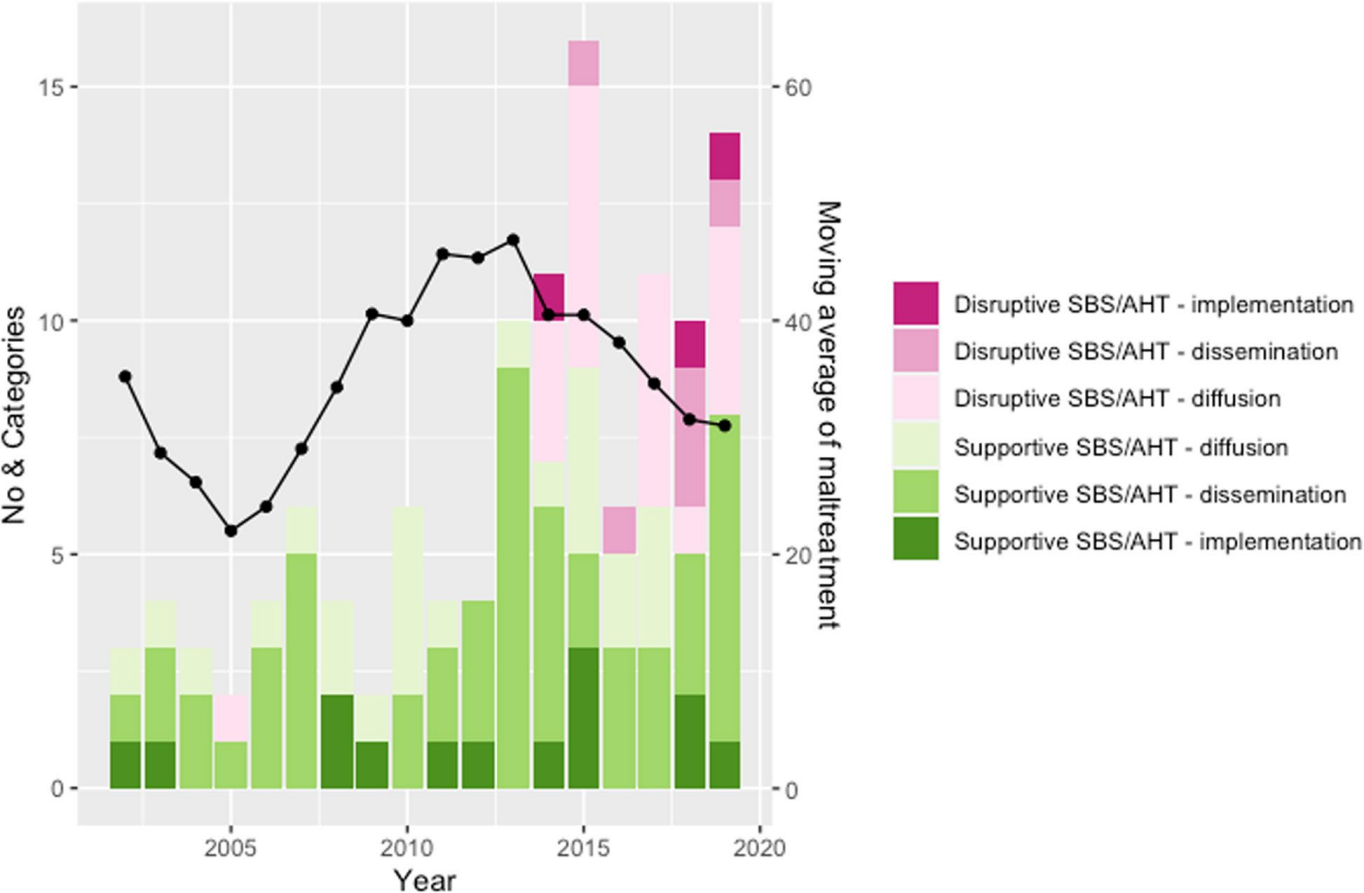
Transfer of supportive or disruptive knowledge on Shaken Baby Syndrome/Abusive Head Trauma by categories diffusion, dissemination and implementation, and moving average of maltreatment diagnoses during the years 2001 to 2019

Table 2 and Fig. 2 show the transfer of knowledge (diffusion, dissemination and implementation) by year in 1990–2019.

**Table 2:** Transfer of knowledge about child abuse, battered-child syndrome, shaken baby syndrome/abusive head trauma (SBS/AHT) in Sweden in the years 1980–2019, categorised as diffusion, dissemination or intervention in accordance with Greenhalgh (2004) and further as being supportive or disruptive to the entities battered-child syndrome or SBS/AHT. Abbreviations: CPT (Child Protection Teams), SPS (Swedish Paediatric Society), SPRS (Swedish Society of Paediatric Radiology), SBU (Swedish Agency for Health Technology Assessment and Assessment of Social Services), SMER (The Swedish National Council on Medical Ethics), SLS (Swedish Medical Society). Footnotes referring to Additional file 3

	1990–2000	2001–2007	2008–2013	2014–2019
**Diffusion (I)**				
**General knowledge**	Research findings, comments^a^	Research findings, overviews, comments^d^	Research findings, overviews, comments^g^	Research findings, overviews, comments^i^
**Battered-child syndrome**	Research findings ^b^	–	–	–
**Supportive SBS/AHT**	Overviews, ^c^	Research findings, overviews, comments^e^	Research findings, thesis, overviews, comments^h^	Research findings, overviews, comments^j^
**Disruptive SBS/AHT**	–	Overview^f^	–	Research findings, comments^k^
**Dissemination (II)**				
**General knowledge**	Textbooks^a^	Textbooks/manuals,, government report^d^	Textbooks /manuals, overviews, conferences, teaching, government report^g^	Manuals, conferences^i^
**Battered-child syndrome**	Textbooks^b^	–	–	–
**Supportive SBS/AHT**	Textbooks/manuals, ^c^	Textbooks/manuals, conferences, teaching, government report^e^	Textbooks/manuals, overviews, conferences, teaching, government report^h^	Overviews, textbooks/manuals, conferences, teaching, government report, comments SPS^j^
**Disruptive SBS/AHT**	–	–	–	Systematic literature review, conferences, seminars SMER, SLS, National Board of Forensic Medicine^k^
**Intervention (III)**				
**General knowledge**	–	Children’s houses, parliamentary inquiry^d^	Children’s houses, government report^g^	Children’s houses, Government supported centre^i^
**Battered-child syndrome**	–	–	–	–
**Supportive SBS/AHT**	–	CPTs, guidelines (SPS), parliamentary inquiry^e^	Child protection taskforce/sub-association of SPS, CPTs, guidelines (SPS & government agencies)^h^	CPTs, guidelines (government agency, SPS & SPRS), Government supported centre^j^
**Disruptive SBS/AHT**	–	–	–	Supreme court acquittals, Positioning National Board of Forensic Medicine^k^

^a^General knowledge 1990–2000. Research: 1991:I;2, 1994:I;4. Comment: 1991:I;1. Textbooks: 1993:II;3, 1999:II;7

^b^Battered-child syndrome 1990–2000. Textbook: 1993:II;3. Research: 1994:I;4

^c^Supportive SBS/AHT 1990–2000. Overview: 1994:I:5, 1996;II:6. Manuals: 1997:II;5. Textbook: 1999:II;7

^d^General knowledge 2001–2007. Research: 2001:I;8. Comment: 2001:I;9. Overview: 2005:I;13. Textbook: 2003:II;10. Manual: 2002:II;9, 2005:II;14. Parliamentary inquiry: 2001:II;8. Government bill: 2002:II:2. Children’s houses: 2001:III;1, 2001:III;2

^e^Supportive SBS/AHT 2001–2007. Research: 2006:I;14, Comment: 2001:I;9. Overview: 2002:I:10, 2005:I;13. Conference: 2004:II;13, 2004:II;14, 2006:II;15, 2007:II;22. Textbook: 2003:II;10, 2007:II;18, 2007:II;19, 2007:II;20, 2007:II;21. Manual: 2005:II;14. 2006–2010:II;16. Parliamentary inquiry: 2001:II;8. Teaching: 2006–2010:II;16, 2006–2010:II;17. Children’s houses 2001:III;2, CPT: 2003:III;3

^f^Disruptive SBS/AHT 2001–2007. Overview: 2005:I;13

^g^General knowledge 2008–2013. Research: 2008:I;16, 2010:1;18, 2010:1;21, 2011:I;22, 2019:I;47. Comment 2010:1;19. Overview: 2010:I;20, 2013:II32, 2013:I;31. Government report: 2010:II;24. Manual: 2011:II;25. Textbook:2011:II;26, 2012:II;28. Conference: 2013:II;34, 2013:II;35. Teaching: 2012:II;28, Guidelines: 2008:III;4, Taskforce/sub-association of SPS:2009:III;6, CPT: 2011;III;7, 2012:III;8

^h^Supportive SBS/AHT 2008–2013. Research: 2008:I:15, 2008:I;16, 2009:I;17, 2010:1;21, 2011:I;22. Overview:: 2010:I;20, 2013:II;30, 2013:II;31, 2013:II;32. Government report: 2010:II;24. Manual: 2011:II;25, 2013:II;37. Textbook:2011:II;26. Conference: 2013:II;34, 2013:II;35, 2013:II;36. Teaching: 2012:II;28. Guidelines: 2008:III;4. Child protection taskforce/sub-association of SPS: 2009:III;6. CPT: 2011;III;7, 2012:III;8

^i^General knowledge 2014–2019. Research: 2018:I;42, 2018:I;43, 2018:I;44, 2018:I;45, 2019:I;46, 2019:I;47, 2019:I;48. Thesis: 2018:I:42. Manual: 2014:II;38. Conference: 2018:II;53. Guidelines: 2014:III:10 (government), 2019:III;18 (SPS). Government supported National Knowledge Centre on Violence against Children: 2015:III;11

^j^Supportive SBS/AHT 2014–2019. Thesis: 2018:I:42. Debate: 2014:1;22. Book review: 2015:I;29. Comment: 2015:I;28, 2016:I;31, 2016:I;32, 2016:I;33, 2016:I;34, 2016:I;35. Overview: 2014:II;40, 2015:I;27, 2019:II;61 & 2019:;62 (guidelines). Editorial/interview SPS: 2017:II;49, 2018:II;52, 2018:II;54. Manual: 2014:II;38. Textbook: 2019:II;55, 2019:II;56. Conference: 2014:II;39, 2015:II;42, 2016:II;45, 2016:II;46, 2016:II;47, 2017:II;50, 2017:II;51, 2018:II;53, 2019:II;58, 2019:II;59, 2019:II;60. Teaching: 2015:II;44. Guidelines: 2014:III:10 (government agency), 2015:III;12, 2018:III;15 (SPR). CPT: 2015:III;13, 2018:III;16. Government supported National Knowledge Centre on Violence against Children: 2015:III;11

^k^Disruptive SBS/AHT 2014–2019. Research: 2015:I;30, 2017:1;41, 2018:I;43, 2018:I;44, 2018:I;45, 2019:I;47, 2019:I;48. Systematic reviews 2016:II;48 (SBU), 2019:II;57. Comment:2014:I;22, 2014:I;23, 2014:I;24, 2014:I;25, 2014:I;26, 2016:I;35, 2017:I;36, 2017:I;37, 2017:I;38, 2017:I;40, 2018:I;41, 2019:I;47, 2019:I;47. Conference: 2014:II;39, 2015:II;42. Supreme court decisions: 2014:III;9, 2018:III;14. Positioning National Board of Forensic Medicine: 2019:III;17

The 1990s was a decade of continuous diffusion and dissemination of general knowledge on child abuse and specifically battered-child syndrome through research, manuals and textbooks. The first mention of SBS came in a short overview in 1994 and SBS was further addressed in a textbook in 1999.

The following two decades were characterised by an increasing transfer of knowledge on child abuse, through diffusion, dissemination and implementation, with a focus on knowledge supportive of SBS (AHT after 2009). Diffusion of new research findings on SBS from the Swedish setting was rare; rather, transfer of knowledge was based on US and UK studies, estimating that close to 100 infants were physically abused annually in Sweden, and only half of them had external signs of abuse, with 1/3 assumed to have been abused by shaking, which allegedly carries the greatest risk of going undetected (Additional file 3 2008:III;4). Dissemination was conducted through textbooks, comments, manuals, conferences (with participation of US and UK representatives) and teaching, emphasising the multidisciplinary diagnostic approach: ‘The radiologist and the ophthalmologist must be prepared to propose in plain text the diagnosis abuse, even though from a clinical view it may seem unlikely.’ (Additional file 3, 2006–2010:II:17). A multitude of interventions supportive of general knowledge on abuse and SBS/AHT were implemented: 1) formation of Children’s Houses (social service leading investigations of abuse in collaboration with healthcare, forensic medicine, police and judiciary); 2) formation of hospital-based Child Protection Teams (CPTs) led by paediatricians (2002–); 3) formation of a sub-association for Child Protection within the Swedish Paediatric Society (2009); 4) guidelines and manuals from Stockholm Healthcare Region and Karolinska Institutet (2008); 5) mental vaccination campaigns against shaking, supported by a national survey in 2011 showing nil shaking. Champions led the implementation activities 2) – 5), as well actively promoting supportive SBS/AHT knowledge by diffusion and dissemination. The years up until 2007 can be characterised by more diffusion and dissemination of knowledge about SBS/AHT, with few implementation activities. The period 2008 to 2013 can be characterised by more intensified implementation.

The period from 2014 can be described as the phase of achieving sustainability in the transfer of knowledge of SBS/AHT, with formations of more CPTs and guidelines issued by the National Board of Health and Welfare (2014) and the Swedish professional societies of paediatric (2018), paediatric radiology (2018) and ophthalmology (2019), However, disruptive knowledge started to emerge. These included acquittals by the Swedish Supreme Court (2014), in which expert witnesses stated that ‘the scientific support for the diagnosis of shaking (AHT, SBS) is unclear’, and the Swedish Supreme Administrative Court (2018), both concluding that certain medical findings indicating shaking violence were not enough for conviction without other findings or circumstances supporting abuse. Foremost in the dissemination of knowledge was the systematic literature review by SBU (2016) concluding: ‘There is insufficient scientific evidence on which to assess the diagnostic accuracy of the triad in identifying traumatic shaking (very low quality evidence).’ The Supreme Court decision in 2014 was questioned and the SBU report was much criticised by representatives of the Swedish Paediatric Society. Diffusion of disruptive knowledge on SBS/AHT was presented by other government agencies and scholars, in seminars, conferences, through new research findings derived from the Swedish setting, and by two paediatricians (2005 and 2017).

Table 3 shows the effect estimates of the incidence rate ratios in the subcategories maltreatment and assault. The change from version 9 to version 10 of the International Classification of Diseases 1997 did not have an effect on the incidence rates of either maltreatment or assault. The inclusion of outpatient specialised care in the registry in 2001 had substantial effects on the incidence rate ratios of the diagnoses of maltreatment and assault, 3.66 (95% CI 1.89–7.78) and 6.93 (95% CI 1.94–44.13). The effect estimate of the period of intensified intervention (2008–2013) was 1.63 (95% CI 1.34–1.98) for maltreatment, compared with the baseline intervention period (2002–2007), while no effect was observed for assault. During the phase of sustainability until obsolescence in 2014–2019 only maltreatment, not assault, decreased compared with in 2008–2013, 0.84 (95% CI 0.71–0.99), while the decrease was more pronounced in 2017–2019, 0.75 (95% CI 0.61–0.92).

**Table 3:** Effect estimates, incidence rate ratio with 95% confidence intervals (CI), of diagnoses of maltreatment or assault during the first year of life among children born 1996–2019 by 1) Swedish version of International Classification of Diseases, 2) inclusion of out-patient specialised care in the NPR (National Patient Register) and 3) intervention effects of transfer of knowledge (diffusion, dissemination, implementation and sustainability until obsolescence) on the diagnosis SBS/AHT (shaken baby syndrome/abusive head trauma)

Year and category		Period	Maltreatment	Assault
			Incidence rate ratio (95% CI)	Incidence rate ratio (95% CI)
**Register**				
ICD version 9	Reference	1996	1	1
ICD version 10	Exposure	1997	1.05 (0.47–2.37)	0.53 (0.11–2.00)
In-patient care only in the NPR	Reference	2000	1	1
Out-patient care added to the NPR	Exposure	2001	3.66 (1.89–7.78)	6.93 (1.94–44.13)
**Transfer of supporting knowledge on SBS/AHT**				
Baseline intervention	Reference	2002–2007	1	1
Intensified intervention	Exposure	2008–2013	1.63 (1.34–1.98)	1.17 (0.84–3.33)
**Transfer of supporting and disruptive knowledge on SBS/AHT**				
Intensified intervention	Reference	2008–2013	1	1
Sustainability until obsolescence (h)	Exposure	2014–2019	0.84 (0.71–0.99)	0.74 (0.53–1.04)
Implementation & sustainability	Reference	2008–2016	1	1
Obsolescence (f)	Exposure	2017–2019	0.75 (0.61–0.92)	0.82 (0.54–1.21)

Table 4 shows risk estimates of incidence rate ratios in the subcategories maltreatment and assault by university healthcare region during 1987–2014. Adjustments by region were statistically significantly different for maltreatment, with two- to five-fold increased incidence rate ratios compared with the reference period 1987–2001, except for the year 2005. Adjusted rate ratios by region for assault showed no differences.

**Table 4:** Effect estimates, incidence rate ratios (95% confidence intervals (CI), adjusted by regional differences and year of on infant diagnoses of maltreatment or assault in Sweden

	Maltreatment		Assault	
Year	Incidence rate ratio (95% CI)	Adjusted incidence rate ratio (95% CI)	Incidence rate ratio (95% CI)	Adjusted incidence rate ratio (95% CI)
1987–2001	1	1	1	1
2002	2.41 (1.68–3.37)	4.36 (2.09–8.39)	1.75 (0.63–3.90)	1.20 (0.00–4.11)
2003	2.92 (2.10–3.96)	3.96 (1.85–7.71)	3.10 (1.47–5.91)	3.48 (0.82–8.58)
2004	1.80 (1.20–2.60)	3.61 (1.65–7.13)	3.32 (1.62–6.20)	3.40 (1.75–6.69)
2005	1.79 (1.19–2.59)	1.69 (0.54–4.11)	3.58 (1.79–6.58)	2.27 (0.00–5.05)
2006	1.87 (1.26–2.66)	3.47 (1.59–6.86)	3.42 (1.71–6.29)	1.64 (0.00–3.55)
2007	2.30 (1.62–3.18)	5.47 (2.90–9.85)	1.30 (0.42–3.07)	1.61 (0.00–4.40)
2008	2.94 (2.15–3.94)	3.11 (1.39–6.25)	2.80 (1.33–5.34)	3.15 (0.00–10.21)
2009	3.17 (2.35–4.21)	7.03 (3.98–12.09)	3.99 (2.13–7.04)	1.56 (0.00–4.52)
2010	3.85 (2.93–5.00)	5.44 (2.94–9.66)	3.13 (1.57–5.76)	1.48 (0.00–4.60)
2011	2.88 (2.10–3.86)	6.26 (3.47–10.94)	2.99 (1.46–5.59)	2.56 (0.00–8.30)
2012	4.59 (3.55–5.87)	7.92 (4.61–13.35)	3.45 (1.77–6.24)	3.54 (0.95–8.63)
2013	3.77 (2.86–4.91)	7.87 (4.58–13.27)	2.94 (1.44–5.50	2.52 (0.00–7.48)
2014	3.28 (2.45–4.32)	5.18 (2.77–9.26)	1.93 (0.80–3.98)	1.47 (0.00–3.55)

Table 5 shows effect estimates of implementation of neuroimaging in investigations. Adjustment by neuroimaging showed no effect on the incidence rate ratios for maltreatment or assault for the years 2007–2014 compared with the years 2005–2006.

**Table 5:** Effect estimates, incidence rate ratios (95% confidence intervals (CI), on infant diagnoses of maltreatment or assault adjusted by implementation of neuro-imaging in investigations for suspected abuse in Sweden

	Maltreatment		Assault	
Year	Incidence rate ratio (95% CI)	Adjusted incidence rate ratio (95% CI)	Incidence rate ratio (95% CI)	Adjusted incidence rate ratio (95% CI)
2005–2006	1			
2007–2008	1.43 (0.90–2.31)	1.26 (0.69–2.31)	0.59 (0.34–0.99)	0.67 (0.31–1.42)
2009–2010	1.92 (1.25–3.02)	1.37 (0.49–3.75)	1.02 (0.65–1.60)	1.43 (0.36–5.68)
2011–2012	2.04 (1.34–3.20)	1.47 (0.55–3.93)	0.92 (0.58–1.46)	1.29 (0.33–4.95)
2013–2014	1.93 (1.26–3.02)	1.45 (0.58–3.50)	0.70 (0.42–1.14)	0.93 (0.27–2.97)

## Discussion

This study shows an increase in maltreatment diagnosis associated with transfer of supportive knowledge of SBS/AHT, while a decline started when disruptive transfer of knowledge began.

How can a two-fold increase of maltreatment diagnoses and then a decline, while assault diagnoses were stable, be interpreted in relation to other countries? Gilbert et al. (2011) performed a comparative analysis of infant diagnosis of maltreatment syndrome or assault (hospital admissions) and homicide and violent deaths in Sweden, England, Western Australia, New Zealand, Manitoba/Canada and the US. The ranges per 100,000 were 11.5 to 118 and 1 to 10.3, respectively [31]. Sweden was at the lower end of the range in both categories. The results of this study up to the year 2000 confirmed these results. However, when adding outpatient diagnoses, substantial increases of both maltreatment and assault diagnoses were noted. The steep increase in maltreatment diagnoses caused Sweden to have incidences comparable to those of England and New Zealand [31].

This increase is remarkable and several interpretations are possible in respect of false positives and false negatives: true increase, hidden cases or overdiagnosis. An actual increase is less likely, as no increase in assault diagnoses was noted and mortality for both maltreatment and assault declined compared with in previous years. A socioeconomic change like immigration, with the mother being foreign-born, had an impact during the years 2008–2014 compared with 1997–2007, but is unlikely to have had a major importance [28]. As mentioned, there was a strong correlation between SBS/AHT criteria and maltreatment diagnosis [28]. Thus, hidden cases or overdiagnosis are likely as alternative explanations. Can the implementation of supportive and disruptive knowledge of the SBS/AHT paradigm into healthcare services support these alternative explanations?

The hidden case hypothesis was claimed to achieve incidences of about 100 per 100,000 through application of the SBS/AHT guidelines. Provided that the SBS/AHT paradigm is an innovation based on solid scientific evidence [6], a large amount of hidden cases of abuse were detected in the Swedish setting, and the experience during 2002–2013 can be considered a success story of implementation of best practice for child protection [8]. The bridging of the knowledge gap for SBS/AHT was characterised by transfer of knowledge through diffusion and dissemination, with reference to US and UK scientific publications, while few research findings from the Swedish setting were presented. Several implementation drivers characterised the process of mainstreaming SBS/AHT knowledge. An organisational readiness [8] was evident, which might have been facilitated by previously implemented knowledge on battered-child syndrome. Organisational champions and leadership [8, 10, 11] and a positive managerial attitude toward changing guidelines regarding SBS/AHT in professional societies, healthcare and government agencies [8] can be identified as core factors. Other crucial factors include the system approach of intervention by creation of CPTs and a specific professional association for child protection [11] and capacity-building through teaching and training [9]. The uneven regional detection rates confirm the finding from New Zealand that formation of CPTs quadrupled the incidence of AHT [28]. The interdisciplinary approach of the intervention [11] might also have been a key factor; in this case facilitated by a standard protocol tool containing diagnostic statements from radiologists and ophthalmologists, irrespective of clinical findings. This can be one reason for high compliance, while protocols that rely on people interacting with other people, to change the behaviours of practitioners, generally have very low success rates [13]. The techno-medicine approach (‘only half of them have external signs of abuse’) might have been essential for the efficacy of implementing SBS/AHT knowledge deviating from the clinical understanding of battered-child syndrome.

The efficacy of the implementation of supportive knowledge of SBS/AHT, *know-how knowledge*, is further indicated by mental vaccination campaigns against shaking, based on hypotheses on the prevention of SBS/AHT. Population surveys in Sweden showed that shaking was reduced from 18% (2006) to almost nil (2011) [28]; however, the increase of maltreatment diagnosis was not impeded during this period. This calls a causal association between shaking and the diagnostic criteria for SBS/AHT into question. There is also a possibility that the mental vaccination campaign was ineffective for prevention of traumatic shaking, since healthcare-seeking of witnessed or admitted shaking may actually have increased during the campaign period [19].

Regarding evidence-based medicine, the overdiagnosis hypothesis is the most likely explanation for the increase of maltreatment diagnosis, supported by the decline observed when disruptive knowledge of the SBS/AHT started to be transferred. Thus, the sustainability of this transfer of knowledge, making the SBS/AHT innovation routine until it reaches obsolescence [8], seems to have been limited, based on the slow decline in the outcome measures during the last 5 years in the study period. The Swedish Supreme Court’s exoneration in 2014 of a father convicted of traumatic shaking might have contributed to this, and was followed by several acquittals in district courts and courts of appeal because of the Supreme Court’s precedence [32]. Similarly, the dissemination of a systematic literature review on the triad of SBS/AHT is likely to have had an impact. In respect of philosophy of science, this challenging of a scientific fact [25] has occurred through transition, with outsiders addressing mainstream questions through new ideas and disruptive findings in a field of science with a decline in publication activity [33]. However, representatives of professional societies have arguied against the Supreme Court’s exoneration and criticised the systematic literature review, even though it emanates from a government agency [34], which usually holds the highest level of credibility among Swedish professionals. Still, the prevailing mainstream thinking seems to be that the diagnostic accuracy of SBS/AHT is a scientific fact, as illustrated by a recent case presentation [35]. This can be interpreted to mean that the construction of SBS/AHT as a scientific fact will take time to change. SBS/AHT is solidly acknowledged worldwide, based on both active elements of knowledge (thought collective) and passive ones (observations) [25], even though new research findings gainsay previous knowledge supportive of SBS/AHT.

Already in 1978, it was discussed that the predictive screening of a protocol for latent markers of child abuse might be low in unselected populations; i.e., misclassification could occur when diagnostic categories, abuse and accidents/diseases, overlapped [36]. The possibility of false positives was again commented on in 1997 by Dr. Sunderland: ‘In the absence of definitive physical signs we may need to profess fallibility. Humility avoids hubris. False diagnoses not only harm children and their families but also devalue the profession.’ [15]. If the scientific foundation of the SBS/AHT paradigm is weak, not evidence-based, the forceful interventions against physical abuse, with a special focus on the SBS/AHT diagnosis, might have caused hundreds of false positive cases of alleged abuse in Sweden alone [37]. This would have had severe consequences for families seeking healthcare for their infants [38]. Hence, in a public health perspective, the findings of this study can be claimed to indicate a situation far from the recommendations of evidence-based child protection from WHO and ISPCAN, to keep false positives at a minimum [1].

Medical practices where best evidence shows no efficacy or harm are common [39]. The half-life of dogma related to surgical practice is estimated at 45 years [40], corresponding to the length of time since the introduction of SBS, although its full implementation came rather late in the Swedish setting. De-implementing obsolete and inappropriate health interventions and procedures is essential for minimising patient harm [39, 41]. The Swedish setting for infant health has experienced two successful de-implementations of harmful practices, strongly supported by professional societies and rapidly executed, first to prevent sudden infant death syndrome [42] and second to prevent coeliac disease [43]. Provided that there were fewer false positive maltreatment diagnoses during the latter study period, the comparatively rather slow decline might be due to obsolescence of clinical guidelines not reconsidered based on disruptive knowledge of SBS/AHT paradigm. Barriers to de-implementation have been recognised among health professionals, leadership and structure, especially if an inappropriate intervention has been delivered for a long period and fear of liability may have been important [41].

From the perspective of implementation science, the findings from this study indicate that the most efficient drivers to change a health practice are the professional societies providing recommendations and guidelines. On the other hand, clinical norms and preferences purportedly in the best interests of the patient may overrule scientific values and new research findings [24]. Hence, lack of readiness of uptake of new research findings to change know-how knowledge among health professionals may be a barrier to evidence-based practice [41].

### Strengths and limitations

A strength of the study was the national coverage of the population registers. The quasi-experimental design of the interrupted time series strengthened internal validity, and presumably balanced external validity. History bias – i.e., events other than the intervention studied occurring during the study period – would be unlikely, thanks to the extensive literature review performed within the study design. Several limitations can be identified. The primary data source, the NPR, did not reach national coverage until 1987, limiting the analysis to the last 33 years. Further, outpatient care was not included in the registry until 2001, hampering comparisons of incidences between the 1990s and more recent years. The ‘real-world’ design might have weakened the selection of timepoints; protocols might have been in use unofficially before their stated year of publication and we had no information on how they were used. Measurement errors could create a bias in either direction, as diagnoses were not checked against clinical records. Hence, the extent to which the increased transfer of general knowledge on abuse has achieved increased detection of true positives cannot be ascertained through this study design. Using diagnoses as the measures of the intervention effect might not be the best way to evaluate transfer of knowledge, as protocols can be circumvented, ignored or selectively employed. Thus, interviews with professionals might be an additional and possibly better tool. However, this fell outside the scope of this study.

## Conclusion

An increase in maltreatment diagnosis was associated with transfer of supportive knowledge about SBS/AHT by diffusion, dissemination and implementation. During the sustainability phase, disruptive knowledge was presented. A decline in diagnosis occurred toward the end of the study period, which might indicate a burgeoning de-implementation process.

## Supplementary Information

**Additional file 1.**

**Additional file 2.**

**Additional file 3.**

## Data Availability

The datasets generated and analysed during the current study are not publicly available due to ethical and legal restrictions prohibiting the sharing of personal data. According to Swedish law and the Swedish Ethical Review Authority, publicly sharing data with personal information is prohibited. Qualified researchers can request the data through the National Board of Health and Welfare at socialstyrelsen@socialstyrelsen.se.
