# Supplementary material for: Transfer of knowledge to diagnose infant abuse and its incidence – a time-series analysis from Sweden
Source: Implement Sci. 2022 Feb 4;17:15. doi: 10.1186/s13012-022-01188-6 (PMC8815122; doi:10.1186/s13012-022-01188-6)
Supplement: Supplementary file 1 — Additional file 1. [file 13012_2022_1188_MOESM1_ESM.docx]

**Annex 1**. A review of how child abuse, with a special focus on infants, was addressed in Swedish scientific publications, textbooks, conferences, government reports and legislation from 1957 to 1986.. Search terms: Sweden, child protection, infants, physical abuse, neglect, battered-child syndrome, subdural haemorrhage, fractures, retinal haemorrhage, out-of-home care.

The first Swedish case report on suspected infant abuse was published in 1957, presenting two infants. Both had bruises and multiple rib fractures, one had a shaft fracture and both had concomitant parental social/psychiatric adversities. In both cases, abuse was denied. Both cases were reported to the local child welfare board (social services). The author referred to Caffey’s publication from 1946 on multiple fractures and subdural haematoma. (1)

In a meeting in 1960 organized by the Swedish Paediatric Society, with the participation of social services and the judiciary, the first guidelines regarding how abuse cases should be assessed and what appropriate measures should be taken were presented (2).

The next case report was published in 1964, presenting six children. Four were infants and one had died. All had external signs of injury, three had subdural haematomas, three had skull fractures, one had metaphyseal lesions, one had a rib fracture, one had clavicle fracture, and all had parental social or psychiatric adversity. Subdural taps were used for diagnosis of subdural haemorrhage. The findings were interpreted as impact injuries. The author referred to Kempe (1962) on battered-child syndrome. (3)

In 1966, the National Board of Welfare published *Advice and instructions related to child abuse*. (4) The publication emphasised the obligation to report suspected abuse to the local child welfare board, which had the right to decide on out-of-home care. According to physicians’ experience, this was the best solution to protect children from a dangerous environment. The publication referred to the state of knowledge, stating that most cases were infants, seldom beyond 3–4 years of age, presenting with injuries to the head, skeleton or with external injuries and external signs of injuries, that parental social or psychiatric adversity were prevalent and that abuse was seldomly admitted. It was also reported that the Ministry of Justice proposed a change in the Parental Code that would mean corporal punishment should in principle be avoided and would also strengthen the wording in the Criminal Code on abuse (4). The *Advice and instructions* were updated in 1970.

Several petitions demanding a survey on the extent of child abuse and the possibilities of prevention were presented to the Parliament in 1965 and the government charged the National Board of Welfare with performing an inquiry. A commission was established, headed by Dr Rolf Zetterström, professor in paediatrics, and with other members representing paediatrics, including Professor Ragnar Berfenstam, including specialists in forensic psychiatry, child psychiatry, sociology and psychology. A report was published in 1969 (5). A literature review of battered-child syndrome was part of the report. For the report, cases of abuse (not neglect) were retrospectively collected from all Swedish hospitals from 1957 througha 1969, with 33 out of 178 departments reporting cases among children aged less than 16 years. They found more cases from metropolitan areas and more cases toward the end of the study period. Out of the 119 cases found, 35 were infants with equal sex distribution. Three children had died, 21 had external signs of injury, 18 had head injuries, six had subdural hematomas, two had skull fractures and thirteen had other fractures. In a sample of 32 children aged less than 3 years who had multiple injuries, 13 had subdural haemorrhages and three had rib fractures. An overrepresentation of parents with social or psychiatric adversity was found. The report stated that the data from 1966 onward were quite reliable, giving a rate of 44 per 100,000 for all cases. This was commented as being considerable lower than what was reported from the U.S. The rate of infant abuse in 1966 can be estimated to 8.5 per 100,000. The recommendations made were improved teaching, transfer of knowledge on child abuse to physicians, nurses and social workers, improvements of systems for notification to local child welfare boards and establishment of a special board at the National Board and Welfare for assessment and follow-up. An appendix to the report consisted of a structured template for notification of suspected child abuse to the local child welfare boards. This could include a report from a forensic doctor if available, and assessment by a social worker. The report further recommended that the local child welfare boards should, if abuse was confirmed, make a decision on out-of-home care, whether police should be notified, and if further psychiatric consultation should be performed, as child abuse was often seen in cases of severe psychopathology in parents. (5)

An editorial in 1967 in the Swedish Medical Journal addressing child abuse described the ongoing government inquiry, emphasizing that many cases had probably previously been undetected, but that the emerging medical knowledge on battered-child syndrome should improve diagnosing. Further, it called attention to the mandatory notification of suspected abuse to local child welfare boards and the improvements made to these boards. It also suggested that social rehabilitation and psychosocial treatment of the parents might be a better option than penal action, not least for the child, in a long-term perspective. (6) The final government report was presented in the Swedish Medical Journal in 1969. (7)

In 1974, a national survey [Children who are abused: A survey of child abuse and a harmful environment growing up] where a questionnaire was sent out by the National Board of Health and Welfare and the foundation Allmänna Barnhuset to the local child welfare boards, requesting data from 1969–1970. A total of 777 cases of abuse were reported, of which 1/3 were among children below age 3 years, 1/3 had indications of physical abuse, of which 1/3 were children below age 1 year (70 per 214,179 = 32.7 per 100,000 (8). In 1975, a commentary in the Swedish Medical Journal summarised the role of social services and healthcare professionals in early detection and prevention (9).

A cross-sectional hospital-based study surveyed diagnosed cases of child abuse during the years 1967 to 1974. Exclusion criteria were that other explanations for the injury were likely and that no negative psychological or social circumstances were present. For these years, 52 cases of child abuse were found; 1/3 (n=16) among children aged less than 1 year. Almost all were examined with full skeletal survey and half went through ophthalmological examination. Examinations bt a specialist in forensic medicine were performed in eight cases. Out of all cases, 2/3 were considered to have minor injuries, such as bruises, while 1/3 had severe injuries, such as fractures, and five had life-threatening injuries. Sex distribution was equal and 12 had a birthweight ≤ 2,500 g. Out of 49, 28 had a previous contact with social services and 19 with the local child welfare board. Thirty-four belonged to the lowest social class. Out of 37 parents assessed for adversity, 33 were classified as having difficulties, such as being prone to giving corporal punishment (n=7), suffering from social adversity (n=9), having been beaten by their own parents (n=6), suffering from psychosis (n=2), or abusing alcohol (n=2). Both social adversity and abuse from own parents were seen in seven of the parents. The authors described the rate in the area as high in a Swedish perspective, but as considerable lower than in reports from the UK and the U.S. (10) A study from the same hospital was performed for the years 1975–1978 (n=103, below age 1 years: n=18), with a doubling in cases found. Most cases had slight injuries, or were anamnestically recorded. The increase was interpreted as a consequence of an extensive education programme in the hospital and town. (11)

In the first edition of the Swedish Handbook in Emergency Paediatrics (1976), child abuse was addressed in one chapter. It underlined the importance of mandatory reporting upon suspicion of child abuse, inconsistencies between the injury and the explanation given by the parents, extensive bruises, multiple fractures, combination of external injuries and fractures, combinations of fractures and internal injuries (pleural haemorrhage, rupture of the spleen, liver, kidneys or intestines), previous care for injuries that could have been abuse-related, or having siblings who had been abused. (12)

At the annual conference of the Swedish Medical Society in 1977, one symposium addressed the right of the child in out-of-home care (only title available) (13). At the same conference, a 10-year report on subdural hygroma in infancy (n=21), encompassing data from two hospitals, was presented (only abstract available). They were diagnosed at age 2 weeks to 6 months using transillumination techniques and pneumoencephalography, having presented with symptoms of feeding problems, irritability and/or delayed psychomotor development. The condition was associated with pregnancy complications (n=9), being born preterm (n=9), being a twin (n=4) and neonatal morbidity (n=11). Physical abuse was not mentioned. (14)

A literature review of child abuse was published in 1978, giving an ecological framing of factors contributing to child abuse and neglect, such as individual, perinatal, parental behavioural and structural factors, also referring to the experiences of Kempe (1971), who had been giving long-term treatment support to parents at home with children (15). At around the same time, a research group conducted a multidisciplinary research project of children exposed to mental, social and physical risks, involving representatives of social work, family therapy, child psychiatry, child psychology, paediatrics and sociology (16).

Child abuse was high on the public agenda in the 1970s. The NGO BRIS (Children’s right in the Society) was founded in 1971 because of the fatal abuse of a 3-year-old girl. It began its efforts with a nation-wide exhibition (17). The children’s book author Astrid Lindgren was also strongly committed to combating child abuse (18).

In 1979, Sweden became the first country to legally ban corporal punishment and emotional humiliation of children. (19) The legislation has been assessed as promoting a normative change in parental behaviour. (20)

**References**

1. Selander P. [Multiple fractures in infants with denied trauma] Swedish Medical Journal. 1957;54:611-6.

2. SOU. [Child abuse - prevention and actions]. Stockholm: Government Offices; 2001:72.

3. Frisk A. [Abused infants]. Swedish Medical Journal. 1964;61:3005-11.

4. Report. [Advice and instructions related to child abuse ] In: [Barnavårdsbyrån], editor. Stockholm: National Board of Health and Welfare; 1966.

5. Zetterström R. [An inquiry into child abuse]. In: Welfare NBoHa, editor. Stockholm: National Board of Health and Welfare; 1969.

6. Editorial. [Child abuse requires active countermeasures]. Swedish Medical Journal. 1967;64(7):652-3.

7. Zetterström R. [What is behind child abuse - and how can it be prevented?]. Swedish Medical Journal. 1969;66:1182.

8. [Children who are abused: A survey of child abuse and a harmful environment growing up] Karlshamn: National Board of Health and Welfare & Allmänna Barnhuset

1974.

9. Selander P. [Children who are abused] Swedish Medical Journal. 1975;72(1-2):42-3.

10. Bergstrand C, Forslund M, Stibner I-B. [Child abuse in Malmö 1967-1974] Swedish Medical Journal. 1976;73(33):2671-7.

11. Bergstrand CG, Forslund M, Stibner IB. Child abuse in Malmö. A comparison between the periods 1967--1974 and 1975--1978. Acta Paediatr Scand Suppl. 1979;275:108-11.

12. Sjölin S. [Social and legal actions, child abuse]. In: Sjölin S, editor. [Emergency Pediatrics]. Stockholm: Almqvist & Wiksell; 1976.

13. Bohman M, von Euler R, Hellström L, Person B, Rutz W, Sverne T, Zetterström R. [The child´s right when being taken into care and custody cases]. In: Medicine TSSo, editor. Annual conference Stockholm: The Swedish Society of Medicine; 1977.

14. Berg E, Bjerre I, Cronqvist S, Strömblad LG. [Chronic subdural haematomas in infants. Review of a 10-year material from Helsingborg and Malmö] Annual Conference Stockholm: The Swedish Society of Medicine; 1977.

15. Lagerberg D. Child abuse: a literature review Acta Paediatr. 1978;67(5):683-90.

16. Gustafsson L, Lagerberg D, Larsson B, Sundelin C. Collaboration in practice. Experiences from a multidisciplinary research project on child abuse and neglect Acta Paediatr Suppl. 1979;275:121-31.

17. [Child abuse in Sweden - a distressing exhibit]. Tidskrift för Sveriges sjuksköterskor. 1971;38(19):16-7.

18. Lindgren A. [Never violence! : speech when receiving German Bookstore peace prize October 22 1978]

1978.

19. [Prohibition of corporal punishment], Law 67 (1979).

20. Ziegert K. The Swedish Prohibition of Corporal Punishment: A Preliminary Report. Journal of Marriage and the Family. 1983;45:917-26.
