# Supplementary material for: Transfer of knowledge to diagnose infant abuse and its incidence – a time-series analysis from Sweden
Source: Implement Sci. 2022 Feb 4;17:15. doi: 10.1186/s13012-022-01188-6 (PMC8815122; doi:10.1186/s13012-022-01188-6)
Supplement: Supplementary file 2 — Additional file 2. [file 13012_2022_1188_MOESM2_ESM.docx]

| **Annex 2.** Diagnoses and injuries coded based on the Swedish versions of International Classifications of Diseases (ICD): ICD-9 (1987–1996), ICD-10 (1997–2019). | | | |
| --- | --- | --- | --- |
|  |  | ICD9 | ICD10 |
| **Maltreatment** | Child maltreatment | E967 |  |
|  | Observation for suspected abuse | - | Z03.8K |
|  | Physical abuse, battered baby syndrome | 995F | T74.1 |
|  | Maltreatment syndrome, unspecified | 995F | T74.9 |
|  | Other maltreatment (incl. physical abuse) by parent | - | Y071A |
|  | Neglect and abandonment | - | Y06 |
|  | Other maltreatment, unspecified | E968.9 | Y07 |
|  | Other diagnoses specifying child maltreatment | 994.2, 994.3, 995.81 | - |
| **Assault** (by) | Drugs, medicaments and biological substances | E962 | X85 |
|  | Corrosive substances | E961 | X86 |
|  | Gases and vapours | - | X88 |
|  | Other specified chemical and noxious substances | E962 | X89 |
|  | Unspecified chemical and noxious substances | E962 | X90 |
|  | Hanging, strangulation and suffocation | E963 | X91 |
|  | Drowning and submersion | E964 | X92 |
|  | Handgun discharge | E965 | X93 |
|  | Rifle, shotgun and larger firearm discharge | E965 | X94 |
|  | Other unspecified firearm discharge | E965 | X95 |
|  | Explosive material | E965 | X96 |
|  | Smoke, fire and flames | E968 | X97 |
|  | Steam, hot vapours and hot objects | - | X98 |
|  | Sharp objects | E966 | X99 |
|  | Blunt objects | - | Y00 |
|  | Pushing from high place | - | Y01 |
|  | Pushing or placing victim before moving object | - | Y02 |
|  | Crashing of motor vehicle | - | Y03 |
|  | Bodily force | - | Y04 |
|  | Sexual assault by bodily force | - | Y05 |
|  | Other means | - | Y08 |
|  | Unspecified means | E968 | Y09 |
|  | Sequelae of assault | E969 | Y87.1 |
|  |  |  |  |
| **External injury to body** |  | 911-917, 919, 922-924 | S10, S11, S20, S30, S31, S40, S50, S60, S70, S80, S81, S90, T09.0, T11.0, T13.0, T13.1, T14.0, T14.1 |
| **Burn** |  | 940-949 | T20 |
| **Head injury and diseases** | Superficial head injury | 872, 873, 910, 918, 920, 921, 925 | S01, S00.8, S00.9, S20.8 |
|  | Brain contusion | 850 | S06.0, S06.1 |
|  | Skull fracture | 800, 801, 803, 804 | S02.0, S02.1, S028, S027, S02.09, S02.9 |
|  | Acute (chronic) subdural haemorrhage | 432B | I62.0 |
|  | Acute traumatic subdural haemorrhage, | 852, 853 | S06.50 |
|  | Epidural haemorrhage |  | S06.4, I62.1 |
|  | Subarachnoidal haemorrhage |  | S06.6, I60 |
|  | Haemorrhagia intracranialis posttraumatica alia sive NUD,  contusione sive laceratione cerebri non indicata / Intracranial posttraumatic bleeding without data on brain contusion or laceration, other or UNS |  |  |
|  | Retinal haemorrhage | 362W | H356 |
| **Fractures** |  |  |  |
|  | Long bone | 812- 813, 819, 820- 824, 827- 828 | S42.2, S42.3, S42,4, S42.7, S42.8, S52, S72, S82 |
|  | Shaft fractures of humerus or femur | 812C,  812D,  821A,  821B | S42.3, S7230 |
|  | Rib | 807A , 819A | S22.3, S 22.4 |
|  | Clavicle | 810A, 810B | S42.0 |
| **Failure-to-thrive** | Failure-to-thrive (starvation) | 783E, 783D | R62.8 |
| **Neglect** | Other affects not otherwise classifiable (e.g., maltreatment of child under 1 year of age) | E904, E904.9 | T74.0 |
