# Supplementary material for: Transfer of knowledge to diagnose infant abuse and its incidence – a time-series analysis from Sweden
Source: Implement Sci. 2022 Feb 4;17:15. doi: 10.1186/s13012-022-01188-6 (PMC8815122; doi:10.1186/s13012-022-01188-6)
Supplement: Supplementary file 3 — Additional file 3. [file 13012_2022_1188_MOESM3_ESM.docx]

**Annex 3.** Transfer of knowledge about child abuse into Swedish child protection from 1990 to 2019. Search terms: Sweden, child protection, infants, physical abuse(not child sexual abuse), neglect, battered-child syndrome, shaken baby syndrome (SBS), abusive head trauma (AHT), subdural haemorrhage, fractures, retinal haemorrhage, out-of-home care. Sources: Publications in medical journals (PubMed)^a^ and grey literature^b^ categorised by knowledge transfer type (Greenhalgh 2004) and whether supportive or disruptive for SBS/AHT: I) Diffusion: Research, overviews, textbooks, comments, interviews, debate, conferences, II) Dissemination: Systematic literature reviews, manuals in textbooks, conference proceedings (symposia), government documents and government agency reports, teaching, III) Intervention: guidelines from government agencies and professional societies, Child Protection Teams (CPTs), Supreme Court decisions.

^a^Including: [Swedish Medical Journal] official journal of The Swedish Medical Association, ^b^Including: [Care] journal for Karolinska Institutet (1983–2002), [The Paediatrician] The Swedish Paediatric Association (2006–2019), Conferences: The Swedish Society of Medicine annual meetings, sections of paediatrics, radiology, neuroscience and ophthalmology (missing 2004). The Swedish Paediatric Society annual meetings (missing 2007, 2011, 2012).

| **Year:**  **Category of transfer; number** | **Title and source** | **General comments, specific comments on reported findings of infant abuse** | **General knowledge on abuse** | **SBS/AHT state of knowledge**  **(supportive or disruptive)** | **Recommended; investigations: skeleton x-ray, fundoscopy, neuroimaging** |
| --- | --- | --- | --- | --- | --- |
| 1990:II;1 | [Child abuse, social and legal actions] Manual^1^ ^2^ | The first edition of Emergency Paediatrics, a manual of recommendations for diagnostics and treatment, was published in 1976. One chapter addressed child abuse and the text was more or less unchanged in the second to fourth editions (1981, 1984, 1990). The text underlined the importance of mandatory reporting when abuse was suspected: 1) when the injuries were not compatible with the parents’ stated cause, 2) Multiple injuries, such as widespread bruises or skin injuries, multiple injuries, fractures, internal injuries (pleural hematoma, spleen, liver, kidneys or rupture of the intestines), 3) Previous healthcare of the child or a sibling for injuries that could have been caused by abuse, 4) Signs of neglect, 5) Delayed care seeking, giving vague and/or contradictory explanations, showing adverse feelings and animosity towards the child. Battered-child syndrome was not mentioned in any edition. | Yes | No | Skeleton |
| 1991:I;1 | [Create a central unit to support abused children] Comment^3^ | Arguing for a central epidemiologic surveillance of all child abuse cases reported to social services. | Yes | No | N/A |
| 1991:I;2 | Intra- and extrafamilial child homicide in Sweden 1971–1980. Cross-sectional study^4^ | National cross-sectional study 1971–1980 reporting homicides (n = 96) of children age < 15 years, mainly characterised by intrafamilial violence with suicide of a parent-perpetrator, 10/47 were found to be mentally ill. A decrease was observed in the last 5 years. | Yes | N/A | N/A |
| 1993:II;3 | [Child abuse and sexual assault] Textbook^5^ | General description of child abuse, mentioning battered-child syndrome. | Yes | No | No |
| 1994:I;4 | [Battered and sexually abused children. Handling routines are based on analysis of medical records] Case series^6^ | Reporting cases of child and sexual abuse (n = 32) during the years 1982–1986 from a university hospital when new guidelines on battered-child syndrome had been implemented: external signs of injury (n = 24), fractures (n = 5), age < 1 years (n = 3). Arguing for close collaboration between paediatrics, children’s psychiatry, forensic medicine, social services and police. | Yes | No | Skeleton |
| 1994:I;5 | [Shaken baby syndrome, severe brain injuries caused by abuse can be detected in the fundus] Overview and case presentation^7^ | First mention of SBS in a Swedish medical journal, overview and case presentations (n = 2) from children’s psychiatry that might have had sequelae from SBS. | No | SBS (supportive) | Yes |
| 1996:I;6 | [Traumatic brain injuries in children, new methods give improved information for prognosis] Overview^8^ | Review of new methods to assess traumatic brain injuries, presenting CT and mentioning rupture of the bridging veins causing subdural haemorrhage due to deceleration and acceleration trauma. | No | SBS  (supportive) | Neuro-imaging |
| 1997:II;5 | [Social and legal actions, child abuse and sexual assault] Book section^9^ | 5^th^ edition of Emergency Paediatrics, now recommending fundoscopy in case of ‘intracerebral injury and head injury’. | Yes | No | Skeleton, fundoscopy added |
| 1998:I;7 | The role of the medical profession in the investigation of child abuse and neglect^e^ Conference paper^10^ | Presentation not available. | - | - | - |
| 1998:II;6 | [Symposium: Child abuse. New knowledge of importance for practice in healthcare] Conference paper^11^ | Presentations not available. | - | - | - |
| 1999:II;7 | [Social and legal actions, child abuse and sexual assault] Textbook^12^ | Review of abuse, including physical abuse, battered-child syndrome (Kempe 1962) and SBS (Caffey 1974), giving guidelines for diagnosis: external findings (bruises, oressure marks, scalding), fractures, intracranial haemorrhages, injuries to liver, spleen, pancreas and kidneys, intestines, | Yes | SBS (supportive) | Yes |
| 2001:II;8 | [Children and abuse, report about corporal punishment and other abuse in Sweden during the late 20^th^ century, Committee Against Child Abuse] Parliamentary inquiry^13^ | The parliamentary inquiry reviewed child abuse, deaths, risk factors, previous inquiries, trends in occurrence. The report stated that adults’ positive attitude towards corporal punishment has decreased from 53% (1965) to 11% (1994), and parents who admitted having grabbed and shaken their child decreased from 49.4% (1980) to 12% (2000), that reports of child abuse for children 0–6 years increased in 1990–1994 but not in 1994–1999, but that healthcare and kindergarten still had great difficulties in reporting child abuse to social services. Based on international comparison, it was stated that child homicide was more uncommon than in the US and UK, SBS was mentioned when referring to the UK. | Yes | SBS  (supportive) | N/A |
| 2001:III;1 | [Child abuse, prevention and actions] Parliamentary inquiry^14^ | The parliamentary inquiry proposed a statutory obligation of cooperation between the local agencies of healthcare, kindergarten, school and police under the leadership of social services in case of child abuse. This was later implemented in the form of Children’s Houses (Barnahus), of which 22 were established in 2004 to 2009, and 32 in total by 2019, covering 77% of all municipalities; social services in collaboration with police, the prosecutor’s office, healthcare, forensic medicine, kindergarten and schools.^15 16^ | Yes | No | N/A |
| 2002:III;2 | [Improved protection to children in vulnerable situations etc.] Government bill to the Parliament^17^ | The government proposed strengthened legal protection for abused children, a mandatory collaboration between healthcare, nursery care and school for child protection under the leadership of social services. The proposal referred to the responsibility of the healthcare sector to detect children in risk environments, to give parental support through antenatal and well-baby clinics, and the role of the Child Protection Team (MIO) at the Karolinska University Hospital as a role model to detect SBS. | Yes | SBS  (supportive) | N/A |
| 2001:I;8 | [Child abuse increases steadily in Sweden - does it correspond to reality?] Observational study^18^ | Trend analysis of the increased rate of police-reported cases of child abuse (0–14 years of age) during 1981–1999. For the age group 0–6 years, no increase in the late 90s was noted. | Yes | No | No |
| 2001:I:9 | [Still, children are being abused, despite new laws and greater awareness on children’s rights – commentary] Commentary^19^ | Commentary to^18^, mentioning and referring to SBS.^7^ | Yes | SBS  (supportive) | No |
| 2002:I:10 | [Abuse of infants, brain injuries and other injuries] Overview^20^ | Overview of physical abuse, including information on SBS. | Yes | SBS  (supportive) | Yes |
| 2002:II;9 | [Learn about child abuse] Manual^21^ | Manual for health professionals and public, one section on child abuse, giving general information and specific information on SBS and the dangers of shaking. | Yes | SBS  (supportive) | Yes |
| 2003:II;10 | [Neglect, abuse and sexual assault] Textbook^22^ | Review of abuse, including information on SBS. | Yes | SBS  (supportive) | Yes |
| 2003:I;11 | [Child abuse most likely cause of severe intracranial trauma with unclear anamnesis in children aged less than 3 years] Overview^23^ | Reporting two case studies from the US (Reece 2000, Hettler 2003) reporting high predictivity for abuse when having intracranial haemorrhage with unclear reported trauma or parents denying abuse. | No | SBS  (supportive) | No |
| 2003:II;11 | [Symposium: Suspected child abuse – why is it so difficult to report] Conference symposia^24^ | Presentations not available. | Possible | Possible | Possible |
| 2003:III;3 | [Guideline on child abuse forces the doctor to act, interview] Interview^25^ | Interview with the initiator of the Child Protection Team (MIO) at Karolinska University Hospital, presenting guidelines for diagnosing SBS/AHT, disseminated nationally upon request, later adopted as official guidelines for the Stockholm healthcare region.^26^ | Yes | SBS/AHT  (supportive) | Yes |
| 2004:II;12 | Plenary lecture Radiology and neuroradiology in physical abuse of infants - strengths and weaknesses. Conference paper^27^ | Presentation not available. | Possible | Likely | Likely |
| 2004:II;13 | Free papers: Inter-sectorial and multi-professional work in child abuse. Experiences from the MIO group, Astrid Lindgren Hospital, Stockholm. Conference paper^28^ | Presentation not available. | Possible | Likely | Likely |
| 2004:I;12 | [Child skull computed tomography – a risk assessment] Comment^29^ | In view of cerebral risk increase (cancer, cognition) from ionising radiation, arguing for CT when infant abuse is suspected, as it can prove cerebral injuries and haemorrhages. | No | SBS  (supportive) | Yes |
| 2005:II;14 | [Children who are abused] Manual^30^ | Sixth edition of Emergency Paediatrics, updated with information on SBS, referring to guidelines from the Karolinska University Hospital (2002). Now added CT/MR to the guidelines. | Yes | No | Neuroimaging added |
| 2005:I;13 | [Swedish child healthcare - a model for other countries, an outstanding development during the last 100 years] Overview^31^ | Review of Swedish paediatric care over 100 years, also referring to the evolving knowledge of SBS, remarking that there were now false positive cases of abuse with those findings. | No | SBS  (supportive & disruptive) | No |
| 2006:II:15 | [Plenary symposium on abusive head trauma] Conference symposium^32^ | Presentation not available. | Possible | Likely | Likely |
| 2006:I;14 | [Child abuse 2006–2007, a national survey] Cross-sectional study^33^ | Survey (N = 1,692) reporting that 18% of parents to infants admitted that they had ‘’pushed, grabbed and shaken’ their child. | Yes | No | No |
| 2006-2010:II;16 | [National graduate medical education course on child maltreatment] Teaching^34^ | Presentations not available. | Possible | Likely | Likely |
| 2006-2010:II:17 | [Physical abuse of small children, one often missed diagnosis. On the immediate care and the clinical result] Teaching^35^ | In the teaching for residents about SBS/AHT, it was stated that ‘[t]he radiologist and the ophthalmologist must be prepared to propose in plain text the diagnosis abuse, even though from a clinical view it may seem unlikely’. | Yes | SBS  (supportive) | Yes |
| 2007:II;18 | [Neglect, abuse and sexual assault] Textbook^36^ | Ibid.^12^ | Yes | SBS (supportive) | Yes |
| 2007:II;19 | [Child ophthalmology] Textbook^37^ | Ophthalmology chapter in paediatric textbook, stating that acceleration/deceleration/rotation of the eye bulb due to shaking caused retinal haemorrhage. | No | SBS (supportive) | Yes |
| 2007:II:20 | [Children who are abused] Textbook^38^ | Textbook about child healthcare, one chapter about child abuse, emphasising for diagnosis external signs of injury, mentioning retinal haemorrhage caused by subdural haemorrhage. | Yes | SBS (supportive) | Yes |
| 2007:II;21 | [Child healthcare – promoting the health of the child] Textbook^39^ | Review and guidelines for detection of child abuse, mentioning intracranial and retinal haemorrhages caused by shaking. | Yes | SBS (supportive) | Yes |
| 2007:II;22 | [Symposium about child abuse: the irresponsible expert testimony] Conference^40^ | Conference, Neuroscience, Karolinska Institutet. Arguing that a SBS diagnosis is certain, the findings cannot have other explanations. Participation of a US attorney and doctor commenting on scholars disputing the SBS diagnosis. | No | SBS  (supportive) | Yes |
| 2008:III;4 | [Regional Clinical Guidelines: When physical abuse of infants and toddlers is suspected]. Guidelines^26^ | Overview and guidelines on SBS from Region Stockholm and Karolinska Institutet, referring to US and UK research on AHT, one of the authors US board certified in subspeciality of child abuse pediatrics. started to be used in 2000–2002, also promoted for national use (still in use 2020). Stating that close to 100 children, less than 1 year, are physically abused annually and half of them have no external signs of abuse, 1/3 abused are through shaking, which carries the greatest risk of going undetected. | Yes | SBS  (supportive) | Yes |
| 2008:III;5 | [Prevention of shaken baby syndrome, report from an educational programme on ‘Mental vaccination’] Report^41^ | A project on mental vaccination against shaken baby syndrome was implemented in part of Region Stockholm 2008–2010. An information package on how to cope with the parental stress when the baby cries inconsolably, the danger of shaking, with a special focus on SBS, was given late in pregnancy, at birth and at well-baby clinics. Most of the parents reported the information as important and a considerable number benefited from the project, which was supported by the Ministry of Social Affairs and Region Stockholm. |  | SBS  (supportive) |  |
| 2008:I;15 | Nurses’ experience in clinical encounters with children experiencing abuse and their parents. Interview study^42^ | Interviews with nurses (n = 11) in emergency wards caring for abused children and their parents. Analytic themes found were: Feelings of ambivalence, nurses’ professionalism and nurses’ care strategies. ‘Participants expressed difficulties in maintaining a professional role in clinical encounters with the parents. The nurses were unhappy in their conflicting roles of both policing (a judging function) and nursing (a caring function).’ | Yes | No | N/A |
| 2008:I;16 | [Information to parents will decrease infant abuse] Interview^43^ | Interview with a neuroradiologist about the first Child Protection Team in Sweden, and ongoing mental vaccination programme. | Yes | SBS  (supportive) | N/A |
| 2009:I;17 | [Evaluation of documentation in potential abusive head injury of infants in a paediatric emergency. Cross-sectional study](https://pubmed-ncbi-nlm-nih-gov.ezproxy.its.uu.se/19389121/)^44^ | Cross-sectional study, presenting children, aged less than 18 months, admitted to a paediatric emergency department and having a head CT (n = 47): SDH (n = 4), documentation of potential child abuse (n = 1). | No | AHT  (supportive) | Yes |
| 2009:III;6 | [Children who are abused, task force for child protection SPS] | Started in 2009 as a task force and in 2015 as a sub-association of the Swedish Paediatric Society, in https://bsfi.barnlakarforeningen.se/om-oss/ | Yes | AHT  (supportive) |  |
| 2010:I;18 | [Few residents in paediatrics are trained in dealing with child abuse] Survey^45^ | Report based on survey from 2009 of paediatric resident training showing that less than half were trained in dealing with child abuse. | Yes | No | N/A |
| 2010:I;19 | [Paediatricians have too little knowledge about child abuse] Commentary^46^ | Arguing for increased training in dealing with child abuse, in particular SBS. | Yes | SBS  (supportive) | Yes |
| 2010:I;20 | [Child abuse - views on prevention in today’s society] Overview^47^ | Overview of child abuse in Sweden and its prevention. | Yes | SBS  (supportive) | No |
| 2010:II;23 | [Paediatric abusive head trauma] Teaching^48^ | Presentations not available. | Possible | Likely | Likely |
| 2010:I;21 | [Child abuse - clinical investigation, management and nursing approach] PhD thesis^49^ | PhD thesis based upon four papers: I & IV^42 44^ and two unpublished (still 2021). II: ‘Children diagnosed as abused - A retrospective study of reporting to social services and the police’ concluding that further educational efforts isre a necessity; III. ‘Neuroradiology of intracranial haemorrhage and associated findings in children younger than 2 years who may be victims of child abuse’ concluding that the neuroradiologist should not only give a description of the findings, but also give an interpretation for further investigation on abuse. | Yes | SBS  (supportive) | Yes |
| 2010:II;24 | [Children exposed to physical abuse] Government report | The National Board of Health and Welfare performed an extensive review of children who were physically abused; shaken baby syndrome described in brief. | Yes | SBS  (supportive) | N/A |
| 2011:II;25 | [Children who are abused] Manual^50^ | Seventh edition of Emergency Paediatrics, updated with information on SBS. | Yes | SBS  (supportive) | Yes |
| 2011:II;26 | [Child abuse] Textbook^51^ | One chapter in a paediatric textbook providing a review of child abuse and SBS. | Yes | SBS  (supportive) | Yes |
| 2011:I;22 | [Physical punishment and violation of children in Sweden – a national survey 2006–2007 & 2011] Reports^52 53^ | A national survey showed that none of the parents (n = 1,358) to infants admitted that they had ‘pushed, grabbed and shook the child’. A finding commented on in 2017 ‘The studies also suggested that Swedish parents have largely ceased to shake small children, particularly infants. It can be hoped that this is the result of national information campaigns that have been implemented over the last 10 years to inform the public of the danger of harming small children by shaking them.’ | Yes | SBS  (supportive) | No |
| 2011:III;7 | Inauguration of Child Protection Team MIO, Stockholm Healthcare Region | Department of Paediatrics, Karolinska University Hospital, official inauguration 1 April 2011.^54^ MIO started their work in 1999/2001 with national dissemination of expertise from the start.^25 43^ | N/A | SBS  (supportive) | N/A |
| 2012:III;8 | Child Protection Team, Health, Care Region Uppsala-Örebro | Department of Paediatrics, Uppsala University Hospital, official inauguration 17 January 2012. | N/A | N/A | N/A |
| 2012:II;27 | [Child Physical Abuse] Conference paper^55^ | Presentation not available. | Possible | Likely | Likely |
| 2012:II;28 | [Children who are abused] Textbook^56^ | Overview about child abuse, clinical investigation and protocol, SBS presented. Reference to guidelines from Stockholm Healthcare Region and Karolinska Institutet.^26^ | Yes | SBS (supportive) | Yes |
| 2012:II;29 | [The physician's role in the multidisciplinary evaluation of child maltreatment] Teaching^57^ | Presentation not available. | Possible | SBS/AHT  (supportive) | Likely |
| 2013:II;30 | [Inflicted head trauma in infants: abusive head trauma] Overview^58^ | Overview of and guidelines for diagnosing AHT. | No | AHT  (supportive) | Yes |
| 2013:I;31 | [How to detect abuse injuries and neglect in children?] Overview^59^ | Overview of how to detect child abuse and specifically AHT. | Yes | AHT  (supportive) | Yes |
| 2013:II;32 | [Child abuse - knowledge, skills and prevention] Overview^60^ | Arguing for improvement of training in child abuse paediatrics and that every paediatric department in the country should have a child protection team. | Yes | AHT  (implicitly supportive) | No |
| 2013:II;33 | [A gradually changing view of child abuse and neglect] Overview^61^ | Overview of child abuse, parental factors and prevention. | Yes | No | no |
| 2013:II;34 | [Child abuse: epidemiological contributions to clinical practice] Conference paper^62^ | Overview of the state of knowledge on child abuse, specifically addressing infant abuse and SBS/AHT. | Yes | AHT (supportive) | Yes |
| 2013:II;35 | [Child protection paediatrics – expertise when a child is suspected to be abused] Conference paper^63^ | Addressing the role of child protection teams. | No | AHT  (implicitly supportive) | No |
| 2013:II;36 | [Physical abuse of the small child, medical and legal challenges: What does the science tell us?] Government Agencies Conference paper^64^ | Swedish, UK and US scholars presenting ‘What does the medical literature tell us?’, ‘Medical controversies in the criminal justice system’ and child protection team and paediatrics | Yes | SBS/AHT (supportive) | Yes |
| 2013:II:37 | [The responsibility of health care for child protection]. Hearing, Swedish Parliament^65 66^. | The Social Affairs Committee of the Swedish parliament had a hearing about the responsibility of health care for child protection. Invited speakers were the founder of the taskforce for child protection, Swedish Society of Pediatrics, a professor from the US also member of the section Child Abuse and Neglect of American Academy of Pediatrics and president of the Ray E Helfer Society and a representative of Save the Children. |  | SBS/AHT supportive |  |
| 2013:II;38 | [Risks of shaking infants] Manual^67^ | Electronic book section providing information to the general public about the dangers of violent shaking causing SBS/AHT and advice to parents for prevention. | No | SBS/AHT (supportive) | Yes |
| 2013:II: 39 | **[**Signs of children being abused]. Electronic book section^68^ | Section in the National Handbook for Child Health Services stating about skull injuries in infants: ‘Infants who are exposed to severe shaking, pounding against a hard surface or a combination of both are at risk of intracranial haemorrhage, damage to the brain and bleeding in the fundus. The child may become unconscious, have seizures and stop breathing … Often, the child also gets broken bone in the ribs, arm, femur and lower legs as a result of the shaking. The risk for permanent brain damage or fatal outcome is great.’ |  |  |  |
| 2014:III;9 | Swedish Supreme Court. Acquittal^69^ | Acquittal of a father accused of SBS/AHT because the only findings supporting AHT were not considered not sufficient for conviction. ‘For it to be considered beyond any reasonable doubt that the mere circumstance that certain physical injuries are at hand that these injuries have been caused by someone in a certain criminal manner, the conclusion must be based on a scientific basis for which there is very strong evidence. At the same time, it must be more or less out of the question that there is any other conceivable explanation for the injuries.’ | N/A | SBS/AHT  (disruptive) | N/A |
| 2014:II; 40 | [Physical signs of child abuse; Physical and behavioural signs of child sexual abuse; Medical child abuse] Manual^70^ | Information to child healthcare services describing findings indicating SBS/AHT. | Yes | SBS/AHT  (supportive) | No |
| 2014:I;22 | [Shaken baby syndrome causes brain injuries to dozens of infants every year] Debate^71^ | Debate article from prominent Swedish paediatricians published in Dagens Nyheter, a leading Swedish newspaper, stating that SBS is causing cerebral injuries in tens of children every year and that the state of knowledge is solid by referring to US and UK references, arguing against a decision in Swedish Supreme Court, citing irresponsible medical witnesses, and demanding that SBU to do a systematic literature review on SBS/AHT. | No | SBS/AHT  (supportive) | No |
| 2014:II; 41 | Meeting, Swedish National Board of Forensic Medicine on SBS/AHT. Conference symposia^72^ | Different perspectives on SBS/AHT – history, philosophical and methodological, child protection teams, forensic pathological, neuropathological and neuroradiological. | No | SBS/AHT (supportive and disruptive) | Yes |
| 2014:III;10 | [Children being abused or at risk of being abused. Guidelines for healthcare and dental care for notification and liability] Guidelines^73^ | Guidelines from National Board of Health and Welfare underlining health professionals’ duty to report child physical abuse, with special attention to AHT (SDH, rib fractures and CMLs). | Yes | AHT (supportive) | No |
| 2014:II:42 | The medical home for maltreated children in the US & Child protection teams in hospital settings. Conference^74^ | Invited lecturer from the section Child Abuse and Neglect of American Academy of Pediatrics, presented at inauguration the Child Protection Team at Queen Silvia´s Children´s Hospital in Gothenburg, and also at two more university hospitals. | - | AHT supportive | - |
| 2014:II;43 | Guest researcher affiliation to Swedish university.^75^ | Uppsala University affiliated as guest researcher, a professor of pediatrics in the US, member of the section Child Abuse and Neglect of American Academy of Pediatrics, also president of the Ray E Helfer Society. He was presented in the application as a world leading researcher in child abuse pediatrics and was expected to contribute to research in child protection at the university, and strengthening the mission of Sweden to have a leading role promoting child protection in Scandinavia and Europe. | - | AHT supportive | - |
| 2014:II; 44 | [Injuries from violence in young children - signs, management and diagnostics] Overview^76^ | Overview on child abuse, evidence-based knowledge, and guidelines on how to detect child abuse, AHT specifically targeted by referring to US scholars and stating an incidence of 20–30 per 100,000 infants according to international literature. | Yes | AHT (supportive) | Yes |
| 2014:I;22 | [Diagnostics of child abuse must be evidence-based] Comment^77^ | Comment to^76^. Questioning the very high positive predictive value of AHT presented based on the defined criteria and the risk of circular reasoning, and proposing case control studies to gain further knowledge. |  | AHT (disruptive) |  |
| 2014:I;23 | [The AHT-model for child protection leads wrong] Comment^78^ | Comment to^76^. Arguing that the negative hermeneutics when neuroradiological and ophthalmological findings indicate AHT and the parents’ history is rejected may lead to false positive cases of abuse. |  | AHT (disruptive) |  |
| 2015:I;24 | [Reasonable to question the AHT model] Comment^79^ | Comment to^76^. Discussing fallacies when diagnosis of abuse is based on the findings of AHT, lack of high-energy trauma, disregarding parents history. Proposing strengthening of family support for chiuld protection. |  | AHT (disruptive) |  |
| 2015:I;25 | [Specificity of findings indicating child abuse a question about etiology] Comment^80^ | Comment to^76^. Final reflection how to come further on etiology of AHT-findings beyond the circular reasoning. |  | AHT (disruptive) |  |
| 2015:I;26 | [Perinatal circumstances and shaking violence] Comment^81^ | Comment to^76^, reasoning about the diagnostic process and potential biases as representative errors, search-satisfying errors and diagnostic momentum, among other conditions that may contribute to findings similar to the AHT criteria. |  |  |  |
| 2014:II; 44 | [Criticism of AHT is unwarranted] Rejoinder/final comment^82^ | Conclusive remarks on the comments, stating that denialism of AHT and irresponsible witnesses presenting alternative hypotheses on findings should not influence child protection, and the solid state of knowledge regarding AHT. |  | AHT (supportive) |  |
| 2015:I;27 | [Paediatric abusive head trauma] Conference^83^ | Presentation not available. | Possible | Likely AHT (supportive) | Likely |
| 2015:I;28 | [The occurrence of shaken baby syndrome is scientifically established] Comment**^84^** | Debate article arguing that AHT is a scientific fact established by 40 years of research, citing a statement from the American Academy of Paediatrics and supporting the ongoing SBU literature review. | No | AHT (supportive) | No |
| 2015:I;29 | [Time to think again and reflect on the AHT-model] Comment^85^ | Arguing about the dangers that AHT should be an exclusion diagnosis that can result in overdiagnosis, citing National Institute of Health (NIH) “The jury is still out and we need to do much more science. We want good, strong science that has been confirmed and verified. |  | AHT  (disruptive) |  |
| 2015:III;11 | [Barnafrid, a national knowledge centre on violence against children] | Barnafrid (2015), a government-supported national centre for knowledge (knowledge centre) on child abuse at Linköping University with the objective to gather the state of knowledge on child abuse among professionals through knowledge transfer, academic courses, and supporting networking of Children’s Houses and Child Protection Teams. Regarding information on AHT, Choudhary (2018) is part of their knowledge database, not the systematic literature review of SBU on SBS. [www.barnafrid.se](http://www.barnafrid.se). | Yes | AHT (supportive) |  |
| 2015:III;12 | [Clinical management overview of child physical abuse] Guidelines^86^ | Internetmedicine ([www.internetmedicin.se](http://www.internetmedicin.se)), a manual for health professionals. First edition in 2012 giving information to healthcare professionals providing an overview of child abuse and specifically addressing SBS/AHT (updated in 2015). | Yes | AHT (supportive) | Yes |
| 2015:I; 30 | [Review of To tell the truth. Robert M Reece *To tell the truth*] Book review^87^ | Book review. | No | AHT (supportive) | no |
| 2015:II; 45 | [Shaken baby, paediatric, perinatal, forensic and psychiatric considerations] Conference^88^ | Symposia addressing shaken baby syndrome and differential considerations. | N/A | AHT (supportive and disruptive) | N/A |
| 2015:II; 46 | [Quick, incest and shaken baby syndrome – ethical issues regarding the role of experts in legal proceedings] Conference^89^ | A seminar by the Swedish National Council on Medical Ethics raising issues regarding the role of experts in legal proceedings, also referring to SBS/AHT proponents’ participating in legal proceedings. | N/A | AHT (disruptive) | N/A |
| 2015:III;13 | Child Protection Team, Healthcare Region South-East | Created in 2010, started as a project in 2015, part of the healthcare organisation in 2018. | Yes | AHT (supportive) | Yes |
| 2015:I; 31 | Circularity bias in abusive head trauma studies could be diminished with a new ranking scale, Research^90^ | Review of case studies regarding precision of AHT diagnosis based on stated AHT criteria, caregivers’ assessments, including multidisciplinary assessments, admissions, family or criminal court proceedings (n = 476). Only 8% of alleged abuse fulfilled the criteria of a new ranking scale, reasonably free of circularity bias: recorded, independently witnessed or confessed abuse. | NA | AHT (disruptive) | NA |
| 2015:II; 47 | [International Course on Child Abuse Paediatrics, InterCAP2015] Teaching^91^ | Presentation not available. | Possible | Likely AHT (supportive) | Likely |
| 2016:II; 48 | [Abusive head trauma: medical and legal perspectives (symposium)] Conference^92^ | Presentation not available. | Possible | Likely AHT (supportive) | Likely |
| 2016:II; 49 | [Paediatric abusive head trauma: experience in Sweden, pre-symposium] Conference^93^ | Presentation not available. | Possible | Likely AHT (supportive) | Likely |
| 2016:II; 50 | [Medical evaluation of suspected inflicted injuries in the young child] Conference^94^ | Presentation not available. | Possible | Likely AHT (supportive) | Likely |
| 2016:II; 51 | [Traumatic shaking: the role of the triad in medical investigations of suspected traumatic shaking] Systematic literature review^95 96^ | The systematic literature review by the Swedish Agency for Health Technology Assessment and Assessments of Social Services (SBU) concluded: 1) There is limited scientific evidence that the triad and, by extension, its components can be associated with traumatic shaking (low quality evidence), 2) There is insufficient scientific evidence on which to assess the diagnostic accuracy of the triad in identifying traumatic shaking (very low quality evidence). | N/A | SBS (disruptive) | N/A |
| 2016:I; 32 | [The SBU-report on shaking violence – far from clinical everyday life] Comment^97^ | Arguing that the SBU report overlooked the clinical experience and the scientific basis for SBS, also mentioning other signs of abuse, such as bruises. | Yes | AHT (supportive) | - |
| 2016:I; 33 | The sowing of unreasonable doubt: lessons learned and setbacks on paediatric abusive head trauma in Swedish courts. Conference paper^98^ | Presentation not available. | Possible | Likely AHT (supportive) | Likely |
| 2017:I; 34 | The way forward in addressing abusive head trauma in infants - current perspectives from Sweden. Comment^99^ | Criticism from officials of the Swedish Paediatric Society regarding the SBU report and its possible consequences. | No | AHT (supportive) | Yes |
| 2017:I; 35 | The new Swedish report on shaken baby syndrome is misleading. Comment^100^ | Arguing for a causal relation between shaking and the components of the triad. | No | AHT (supportive) | Yes |
| 2017:I; 36  . | Extensive shaken baby syndrome review provides a clear signal that more research is needed. Comment^101^ | Acknowledging the SBU report as a classic paper, has criticisms, discussing the challenges of research and clinical practice. | No | AHT (supportive and disruptive) | Yes |
| 2017:I; 37 | Authors’ overarching reply to all the responses received to the systematic literature review on shaken baby syndrome. Comment^102^ | The expert team of the SBU report responded to criticism of the SBU report. |  | AHT (disruptive) |  |
| 2017:I; 38 | Conflicts of interest issues. Response to Lucas et al. Comment^103^ | The expert team of the SBU report responded to criticism of the SBU report |  |  |  |
| 2017:I; 39 | What are acceptable conclusions? Response to Dr Ludvigsson. Comment^104^ | The expert team of the SBU report responded to criticism of the SBU report |  |  |  |
| 2017:II;52 | [Shaken baby syndrome – an issue that concerns us all] Editorial^105^ | The board of the Swedish Paediatric Society criticised the SBU report, supporting their sub-association Child Protection (Children who are abused) in managing the matter, urging the National Board of Health and Welfare to authorise national guidelines. |  | AHT (supportive) |  |
| 2017:II; 53 | [Infant abuse, investigation in case of suspicion of abusive head trauma. Work model based on proposals for guidelines advocated by the sub-association Child Protection & the SBU report – how everyday clinical practice is affected] Conference^106 107^ | Presentations not available. | Possible | Likely AHT (supportive) | Likely |
| 2017:I;40 | [Shaken baby syndrome under scrutiny: a new orientation of the entire procedure is required ] Comment^108^ | Comment in the journal of Swedish paediatricians, arguing for reconsidering the diagnostic process for infant abuse after the publication of the SBU report, not only relying on statements from radiologists or ophthalmologists. | Yes | AHT (disruptive) | Yes |
| 2017:II; 54 | Unreasonable doubt: how Swedish courts and agencies are undermining the science in abusive head trauma cases. Conference^109^ | Presentation not available. | Possible | Likely AHT (supportive) | Likely |
| 2017:I;41 | National study shows that abusive head trauma mortality in Sweden was at least 10 times lower than in other Western countries. Research article^110^ | National study of all deceased infants in 1994 to 2013 (n = 733), scrutinised regarding possible cases of AHT (n = 12), with 8 was diagnosed as AHT, concluding that the risk of unreported fatal AHT was low. The incidence was at least 10 times lower than in other Western countries; further, there may have been cases misdiagnosed as AHT. | N/A | AHT (disruptive) | N/A |
| 2018:III;14 | Swedish Supreme Administrative Court. Decision^111^ | Father accused of SBS/AHT regained custody: Only triad findings not sufficient for a decision on out-of-home care, ‘As regards suspicion of physical abuse of a child, when the judgment is based only on the existence of certain symptoms in the child, a prerequisite for mandatory interventions should be that there is clear scientific support for a causal connection between the medical findings and the required violence […] If there is no clear scientific support for a causal connection, the absence of other possible explanations for the injuries cannot in itself be considered to make it likely that the injuries have been caused by violence.’ | N/A | AHT (disruptive) | N/A |
| 2018:I;41 | [The conclusions of the SBU report about diagnostics of shaken baby syndrome remain] Comment^112^ | The expert team of the SBU report arguing for the validity of conclusions of the SBU report. | No | No |  |
| 2018:II; 55 | [Rejoinder by Swedish Paediatric Society] Comment^113^ | Rejoinder from the Swedish Paediatric Society acknowledging the responsibility of the paediatrician in collaboration with other specialists to perform a meticulous investigation in collaboration with social services and the police, agreeing with the SBU expert team that more research is needed, and stating that they were negotiating with the National Board of Health and Welfare to provide new guidelines. No arguing against the SBU report. | Yes | No |  |
| 2018:II; 56 | [Collaboration and investigation of infant abuse] Conference^114^ | Overview of infant abuse, especially SBS/AHT, case presentations. | Yes | AHT (supportive) | yes |
| 2018:II; 57 | [Paediatricians: Uncertainty has spread after the SBU report/] Interview^115^ | Interview with the president of the Swedish Paediatric Society alerting the National Board of Health and Welfare of ‘…concern about the uncertainty among those employed in healthcare, social services and the judiciary that has spread since the SBU report was published’. |  | AHT (supportive) |  |
| 2018:III;15 | [Guidelines for radiology during investigation of suspected child abuse] Guideline^116^ | Guideline for abuse investigations from the Swedish Society of Paediatric Radiology recommending that the X-ray referral should clearly state that abuse is suspected (both in writing and verbally), and that the radiologist should describe the findings clearly and systematically. | No | No | yes |
| 2018:I;42 | Health sector and community response to child maltreatment in Sweden and in a European context. PhD thesis^117^ | PhD thesis based upon four published studies: Disclosure of child physical abuse and perceived adult support among Swedish adolescents.^118^ Factors influencing the prosecution of child physical abuse cases in a Swedish metropolitan area.^119^ Paediatric approaches to child maltreatment are subject to wide organisational variations across Europe.^120^ Childhood death rates declined in Sweden from 2000 to 2014, but deaths from external causes were not always investigated.^121^ | Yes | No | No |
| 2018:III;16 | Child Protection Team Healthcare Region South | Official inauguration 19 November 2018. | Yes | Yes | Yes |
| 2018:I;43 | Infant abuse diagnosis associated with abusive head trauma criteria: Incidence increase due to overdiagnosis? Research^122^ | Register-based national study of infants born 1987-2014 showing an increase of maltreatment diagnosis (assault not included); diagnosis was strongly associated with SBS/AHT criteria, such as subdural haemorrhage, rib fracture, retinal haemorrhage, skull fracture and long bone fracture. |  | Disruptive findings |  |
| 2018:I;44 | Epidemiology of subdural haemorrhage during infancy: A population-based register study. Research^123^ | Register-based national study of infants born 1997-2014 with subdural haemorrhage (SDH): non-birth related (n = 251), short falls reported (n = 104); risk factors for non-traumatic SDH were being male, born preterm and small-for-gestational age, as for those with SDH and abuse (n = 43). | No | Disruptive findings | N/A |
| 2018:I;45 | Metabolic bone disease risk strongly contributing to long bone and rib fractures during early infancy: A population register study. Research^124^ | Register-based national study of infants born 1997-2014 with fractures (n = 4,663): 71% had fall accidents. Metabolic bone disease risk factors prominent in case of fracture in long bone and ribs up to 6 months of age. Vitamin D deficiency and rickets were risk factors for fractures in long bone and ribs. Abuse (n = 105) had overrepresentation among those preterm, multiple births and small-for-gestational age. | No | Disruptive findings | N/A |
| 2019:II; 58 | [Inflicted head injury in infants] Book section^125^ | Textbook about child neurology, one chapter presenting AHT. |  | AHT (supportive) | Yes |
| 2019:II; 59 | [Children who are abused] Manual^126^ | Eight edition of Emergency Paediatrics, a manual of recommendations in case of suspected abuse as regards clinical handling, investigation, diagnostics, calling social services, recommendation to call the police, blocking access to electronic medical records for the parents. | No | No | Yes |
| 2019:II; 60 | With what certainty can it be claimed that rib fractures or classical metaphyseal lesions in infants are attributed to physical abuse? A systematic literature review^127^ | MSc thesis, systematic literature review concluding: There is *limited scientific evidence* that CML can be caused by abuse. There is *insufficient scientific evidence* on which to determine the diagnostic accuracy of CML being caused by abuse. There is *insufficient scientific evidence* that rib fractures can be caused by abuse and therefore also *insufficient scientific evidence* on which to determine the diagnostic accuracy of rib fractures being caused by abuse. | No | Disruptive findings |  |
| 2019:III;17 | Protocol from the National Board of Forensic Medicine^128^ | ‘Unlike healthcare, the National Board of Forensic Medicine, as an authority that requires impartial expert statements, has listened to the Supreme Court, the Supreme Administrative Court and SBU, as regards both the triad and fractures, recommending more nuanced and cautious assessments of infants with the triad or fractures.’ |  | Disruptive positioning |  |
| 2019:II; 61 | Confronting Child Abuse Challenges in the Wake of the Swedish Agency for Health Technology Assessment (SBU) Report on Abusive Head Trauma (AHT). Conference^129^ | Presentation not available. | Possible | Likely | Likely |
| 2019:I;46 | Childhood death rates declined in Sweden from 2000 to 2014, but deaths from external causes were not always investigated. Research^121^ | Analysis from Swedish death cause register for children 0–17 years of age from 2000-2014; a quarter of deaths were from external, ill-defined or unknown causes. Proposal for systematic, interagency death reviews that might yield information that could prevent future deaths. | N/A | No | N/A |
| 2019:I;47 | Medical diagnoses among infants at entry in out-of-home care: A Swedish population-register study. Research^130^ | Register-based national study of children born 1997-2014 and in out-of-home care as infants: 2.4% had diagnoses compatible with SBS/AHT criteria, such as subdural haemorrhage, retinal haemorrhage, rib fractures or long bone fractures (n = 182). The incidence increased over time. | Yes | N/A | N/A |
| 2019:I;48 | Population-based register study of children born in Sweden from 1997 to 2014 showed an increase in rickets during infancy. Research^131^ | Register-based national study of children born 1997 to 2014 reporting an increased incidence of rickets, vitamin D deficiency, vitamin D-resistant osteomalacia rickets and unspecified disorders of bone density and structure (n = 273). Two cases had a previous diagnosis of abuse. | N/A | Disruptive findings | N/A |
| 2019:II; 62 | The SBU Controversy – Is It *Really?* Conference^132^ | Presentation not available. | Possible | Likely | Likely |
| 2019:II; 63 | A Flawed Report out of Sweden: Can the Institution Undermine Physicians and their Work? Conference paper^133^ | Presentation not available. | Possible | Likely | Likely |
| 2019:III;18 | [Medical routine, management of suspected infant abuse]. Guidelines^134^ | Swedish Paediatric Society presenting guidelines for investigation, action and follow-up. Guidance on when infant abuse should be suspected, SBS/AHT not mentioned. Protocol including skeleton x-ray fundoscopy, neuroimaging, no guidance on radiologic or ophthalmologic findings indicating abuse. Securing the safety of the child, calling social services and the police, blocking access to electronic medical records for the parents. | No | AHT supportive | Yes |
| 2019:II; 64 | [Retinal haemorrhage in violence to the head in young children. Proposal for clinical management support] Overview & guidelines^135^ | Overview of AHT, providing state of knowledge that specific retinal haemorrhage is diagnostic for SBS/AHT, and clinical guidance. | No | AHT supportive | Yes |
| 2019:I; 49 | [We look forward to the authors presenting their own fundus images] Comment^136^ | Arguments for the solidity of the SBU report, and asking the authors to present their own experiences. | - | Disruptive reasoning | - |
| 2019:I; 50 | [Haemorrhage due to vitreoretinal traction – truth or hypothesis?] Comment^137^ | Scrutinising the hypotheses that vitreoretinal traction or increased intracranial pressure causes retinal haemorrhage. | - | Disruptive reasoning | - |
| 2019:II; 65 | [Concluding remarks regarding AHT: Replies show the need for ophthalmologists’ competence in AHT] Comment^138^ | Final remarks arguing for the need of ophthalmologists in detection of AHT. | - | Yes | - |

**References**

1. Sjölin S. [Social and legal actions, child abuse]. In: Sjölin S, ed. [Emergency Pediatrics]. Stockholm: Almqvist & Wiksell 1976.

2. Ludvigsson J. [Social and legal actions, child abuse and sexual assault] In: Sjölin S, Larsson A, ed. [Emergency Pediatrics] Fourth ed. Stockholm: Almqvist & Wiksell, 1990.

3. Mjönes S, Janson S. [Create a central unit to support abused children] *Swedish Medical Journal* 1991;88(30-31):2503-4.

4. Somander L, Ramner L. Intra- and extrafamilial child homicide in Sweden 1971-1980. *Child Abuse Negl* 1991;15:45-55.

5. Sandberg N, Elander G. [Child abuse and sexual assault] In: Sandberg N, Elander G, ed. [Paediatrics] Stockholm: Liber 1993.

6. Svedin G, Gustafsson A. [Battered and sexually abused children. Handling routines are based on analysis of medical records]. *Swedish Medical Journal* 1994;91(4):277-32.

7. Janson B. [Shaken baby syndrome, severe brain injuries caused by abuse can be detected in the fundus] *Swedish Medical Journal* 1994;91:491-99.

8. Christerson S. [Traumatic brain injuries in children, new methods gives improved information for prognosis] *Swedish Medical Journal* 1994;35:2951-56.

9. Ludvigsson J, Sundelin C. [Social and legal actions, child abuse and sexual assault] In: Larsson A, ed. [Emergency Pediatrics] Fifth ed. Falköping: Liber 1997.

10. Meadow R. The role of the medical profession in the investigation of child abuse and neglect. In: Medicine Sso, ed. Pediatric section, annual meeting of Swedish Society of Medicine. Stockholm: The Swedish Society of Medicine, 1998.

11. Borre B, Broberg A, Sundelin C, Sundell K. [Symposium: Child abuse. New knowledge of importance for practice in health care]. In: pediatrics So, ed. Annual meeting of Swedish Society of Medicine. Stockholm: Swedish Society of Medicine, 1998.

12. Svedin G. [Neglect, abuse and sexual assault] In: Lagercrantz H, ed. [Paediatric medicine]. Lund: Studentlitteratur 1999:180-96.

13. Janson B. [Children and abuse, report about corporal punishment and other abuse in Sweden during the late 20th century, Committee Against Child Abuse] In: Government, ed. Stockholm: Government, 2001.

14. Hedkvist Petersen E. [Child abuse, prevention and actions]. Stockholm: Government, 2001.

15. Kaldal A, Disen C, Beije J, Diesen E. [Assessment of Children´s Houses]. Stockholm: Department of Law, 2010.

16. Korhonen L, Ekerfelt C. Assessment of Children´s House, final report. Linköping: [Barnafrid, National Knowledge Center Against Child Abuse (nationellt centrum för kunskap om våld mot barn)], 2019.

17. [Government proposition to the Parliament: Improved protection to children in vulnerable situations etc.]. 03:53, 2002.

18. Nilsson C, Horgby K, Borres M. [Child abuse increase steadily in Sweden - does it correspond to reality?] *Swedish Medical Journal* 2001;98:2298-301.

19. Janson S. [Still, children are being abused, despite new laws and greater awareness on children´s rights - commentary] *Swedish Medical Journal* 2001;98:2290-91.

20. Flodmark O. [Abuse of infants, brain injuries and other injuries] *[Vård, Karolinska Institutet]* 2002(1):23-31.

21. Svedin G. [Learn about childabuse] In: Lindberg T, ed. growingpeoplese: growingpeople.se, 2002.

22. Svedin G. [Neglect, abuse and sexual assault]. In: Hanséus K, Lagercrantz H, , ed. [Paediatric Medicine] Lund: Studentlitteratur 2003.

23. Janson S. [Child abuse most likely cause of severe intracranial trauma with unclear anamnesis in children aged less than 3 years] *Swedish Medical Journal* 2003;100(25):2205.

24. Flodmark O, Carlberg M, Eriksson L, Kjellgren C, Svensson P-J. [Symposium: Suspected child abuse – why is it so difficult to report] In: Sections for pediatrics n, ophtalmology, ed. Annual meeting of Swedish Society of Medicine. Stockholm: Swedish Society of Medicine 2003.

25. Hedbäck S. [Guideline on child abuse forces the doctor to act, interview] *Swedish Medical Journal* 2003;100(10):822-3.

26. Flodmark Oe, Hirsch G, Tingberg B, Svensson P-J, Gustavsson B, Otterman G, Kaiser S, Martin H, Jahnke M, Erixon M, Rydh B, Jacobson L, Åström E, Almqvist P. [Regional guideline: care programme on suspicion of physical abuse of infant and toddlers] Stockhom: Stockholm County Council and Karolinska Institutet, 2008.

27. Flodmark O. Plenary lecture: Radiology and neuroradiology in physical abuse of infants - strengths and weaknesses. Third Congress Nordic Association for Prevention of Child Abuse and Neglect Turku: NFBO, 2004.

28. Flodmark O. Free papers: Intersectorial and multiprofessional work in child abuse. Experiences from the MIO-group, Astrid Lindgren Hospital, Stockholm. Third Congress. Turku: Nordic Association for Prevention of Child Abuse and Neglect, 2004.

29. Flodmark O, Hall P, Ingvar M [Child skull computed tomography – a risk assessment] *Swedish Medical Journal* 2004;101(8):706-07.

30. Ludvigsson J, Stinzing G. [Children who are abused] In: Larsson A, Norgren S, Lindquist B, ed. [Emergency Paediatrics]. 6th ed. Stockholm: Liber 2005.

31. Zetterström R. [Swedish child health care - a model for other countries, an outstanding development during the last 100 years] *Swedish Medical Journal* 2005;102(23):1808-14.

32. Otterman G. [Plenary Symposium on Abusive Head Trauma]. Annual meeting. Stockholm: Swedish Paediatric Society, 2006.

33. Svensson B, Långberg B, Janson S. [Child abuse 2006-2007, a national survey] Stockholm, 2007.

34. Otterman G. Course Director: graduate medical education course on child maltreatment (2006-2010). Stockholm: Ipuls AB, 2006.

35. Flodmark O. [Physical abuse of small children, one often missed diagnosis. On the immediate care and the clinical result. National graduate medical education course on child maltreatment] Stockholm: Ipuls AB, 2006.

36. Svedin G. [Neglect, abuse and sexual assault]. In: Lindberg T, Lagercrantz H, ed. [Paediatric Medicine] Lund: Studentlitteratur 2007.

37. Jacobsson L. [Child ophthalmology] In: Lindberg T, Lagercrantz H, ed. [Paediatric medicine] Lund: Studentlitteratur 2007.

38. Lagerberg D. [Barn som far illa] In: Hagelin E, Magnusson M, Sundelin C, ed. [Barnhälsovård] Fourth Edition ed. Stockholm: Liber 2007.

39. Lagerberg D. [Children who are abused]. In: Hagelin E MM, Sundelin C ed. [Child health care – to promote the health of the child]. Fourth ed. Stockholm: Liber 2007.

40. Flodmark O, Åström E, Blennow M, Almqvist P, Much J, Ny M, Hadding-Wiberg G, Reece R, Holmgren B, Bergström M. [Symposium about child abuse: the irresponsible expert testimony] In: Neuroscience, ed. Stockholm: Karolinska Institutet, 2007.

41. Tegern G, Tinghög P, Flodmark O. [Prevention of shaken baby syndrome, report from an educational programme on "Mental vaccination"]. Stockholm: Karolinska Institutet & Linköping University, 2012:134.

42. Tingberg B, Bredlov B, Ygge BM. Nurses' experience in clinical encounters with children experiencing abuse and their parents. *J Clin Nurs* 2008;17(20):2718-24. doi: 10.1111/j.1365-2702.2008.02353.x [published Online First: 2008/09/24]

43. Åhs V. [Information to parents will decrease infant abuse, interview with Olof Flodmark]. *Pediatrician* 2008(4):27-28.

44. Tingberg B, Falk AC, Flodmark O, et al. Evaluation of documentation in potential abusive head injury of infants in a Paediatric Emergency Department. *Acta Paediatr* 2009;98(5):777-81. doi: 10.1111/j.1651-2227.2009.01241.x [published Online First: 2009/04/25]

45. Mårtensson T, Janson S. [Few residents in pediatrics are trained about child abuse] *Swedish Medical Journal* 2010;107(35):1996-198.

46. Flodmark O. [Pediatricians have too little knowledge about child abuse] *Swedish Medical Journal* 2010;107(35):1992.

47. Janson S. [Child abuse - views on prevention in today´s society] *Pediatrician* 2010(4):9-10.

48. Otterman G. [Pediatric Abusive Head Trauma]. Stockholm: Karolinska Institutet, 2010.

49. Tingberg B. Child abuse - clinical investigation, management and nursing approach. PhD Thesis, Karolinska Institutet, 2010.

50. Ludvigsson J, Stinzing G. [Children who are abused]. In: Norgren S, Ludvigsson J, Norman M, ed. [Emergency paediatrics]. 7th ed. Stockholm: Liber AB 2011.

51. Skanse B, Jägervall M. [Child abuse]. In: Widegren M, ed. [Ped]. Stockholm: Liber AB 2011.

52. Janson. S. LB, Svensson B. [Physical punishment and violation of children in Sweden – a national survey 2006-2007 & 2011] Stockholm: Allmanna Barnhuset and Karlstad University, 2007 & 2011.

53. Jernbro C, Janson S. [Physical punishment and violation of children in Sweden – a national survey 2016] In: The Children’s Welfare Foundation S, ed.: The Children’s Welfare Foundation, Sweden, 2017.

54. Flodmark O, Fridén B, Janson S, Svedin G, Ingvar M, Tingberg T, Rosengren L, Reinfeldt F. [Inauguration of the Child Protection TeamMio at Astrid Lindgrens children´s hospital]. The child protection team Mio, Astrid Lindgrens children´s hospital. Stockholm: Karolinska University Hospital, 2011.

55. Svensson P, Otterman G. [Child Physical Abuse, graduate seminar]. Annual Conference. Linköping: Swedish Surgical Society, 2016.

56. Janson S. [Children who are abused]. In: Lagercrantz H, ed. [Paediatric medicine]. Lund: Studentlitteratur 2006.

57. Otterman G. [The Physician's Role in the Multidisciplinary Evaluation of Child Maltreatment, co-course director]. Uppsala: Uppsala University, 2012.

58. Otterman G. [Inflicted head trauma in infants: Abusive Head Trauma] *Pediatrician* 2013(5):8-9.

59. Tindberg Y, Borgström P. [How to detect abuse injuries and neglect in children?] *Pediatrician* 2013(5)

60. Sarkadi A, Lucas S, Otterman G. [Child abuse - knowledge, skills and prevention]. *Pediatrician* 2013(5):10-11.

61. Köhler M, Janson S [A gradually changing view of child abuse and neglect]. *Pediatrician* 2013(5):6-7.

62. Janson S. [Child Abuse: epidemiological contributions to clinical practice]. In: Society P, ed. Annual meeting of Swedish Paediatric Society. Karlstad, 2013.

63. Otterman G. [Child protection pediatrics – excellence when a child is suspected to be abused]. Annual meeting of Swedish Paediatric Society

Karlstad: Swedish Paediatric Society, 2013.

64. Otterman G, Maguire S, Greeley C, Janson S, Kaldal A. [Physical abuse of the young child, medical and legal challenges: What does the science tell us?] Uppsala: Swedish Crime Victims Compensation and Support Authority in collaboration with the Swedish Paediatric Society and Uppsala University Children's Hospital 2013.

65. Otterman G. Curriculum Vitae: International Society for the Prevention of Child Abuse and Neglect (ISPCAN), 2019.

66. [The responsibility of health care for child protection, Anders W Jonsson (chair), Howard Dubowitz, Martin A Finkel, Gabriel Otterman, Anna Kaldal, Åsa Landborg]. Social Affairs. Stockholm: Swedish Parliament, 2013.

67. Erlingson M, Tingberg B. [Risks of shaking infants] In: Erlingson M, ed. 1177 Vårdguiden. 2013-12-11 ed. Stockholm: Inera AB, 2013.

68. Lucas S. [Signs that children are being abused]. In: Åkerman A, ed. The National Handbook for Child Health Services

Stockholm: Inera AB, 2013.

69. [Decision case B3438 -12, 2014-10-16] Stockholm: Swedish Supreme Court, 2014.

70. Lucas S, Otterman G, Köhler M. [Physical signs of child abuse; Physical and behavioral signs of child sexual

abuse; Medical child abuse]. In: Åkerman E, ed. The National Handbook for Child Health Services. Stockholm: Inera AB, 2014.

71. Otterman G, Ludvigsson J.F., Söder O, Svedin C.G, Köhler M, Jansson S, Lucas S. [Shaken baby syndrome causes brain injuries to dozens of infants every year] *Dagens Nyheter* 2014 15 February.

72. Olofsson T, Thiblin I, Otterman O, StrayPedersen A, Squier W, Mack J, Anderson J. Meeting of the Swedish National Board of Forensic Medicine on SBS/AHT Meeting of the Swedish National Board of Forensic Medicine on SBS/AHT. Uppsala: Swedish National Board of Forensic Medicine, 2014.

73. Danon A. [Children being abused or at risk of being abused. Guidelines for health care and dental care for notification and liability]. Stockholm: National Board of Health and Welfare 2014.

74. Christian CW. Curriculum Vitae, 2014.

75. Lucas S. [Application to invite Howard Dubowitz as guest researcher at Uppsala University ]. Uppsala: Uppsala University, 2013.

76. Otterman G, Tindberg Y. [Injuries from violence in young children - signs, management and diagnostics] *Swedish Medical Journal* 2014;111:CZYR.

77. Thiblin I. [Diagnostics of child abuse must be evidence-based]. *Swedish Medical Journal* 2014;111:C9SM.

78. Högberg G. [AHT model in child protection is deceiving]. *Swedish Medical Journal* 2014;111(C9UC)

79. Högberg G. [Reasonable to question the AHT model]. *Swedish Medical Journal* 2015;112:DADL.

80. Thiblin I. [Specificity of child abuse diagnosis an issue of etiology]. *Swedish Medical Journal* 2015;112:DACX.

81. Högberg U. [Perinatal circumstances and shaken baby syndrome] *Swedish Medical Journal* 2015;112:DAAM.

82. Otterman G, Tindberg Y. [Criticism of AHT is unwarranted]. *Swedish Medical Journal* 2014;111:C9ST.

83. Otterman G. [Pediatric Abusive Head Trauma] Annual Conference Uppsala: Swedish National Society for Anaestesesia and Intensive Care, 2015.

84. Ludvigsson JF, Steinwall Larsen S, van Agthoven G, Borgström P, Ränfors J, Gold E, Wide P, Janson S, Köhler M, Lucas S, Holmlund U. [The occurrence of shaken baby syndrome is scientifically established] *Swedish Medical Journal* 2015;112:DE6U.

85. Högberg G, Högberg U, Norlander K, Davidsson L [Time to think again and reflect on the AHT-model] *Swedish Medical Journal* 2015;112:DFMM.

86. Ludvigsson JF, Otterman G, Janson S. [Clinical management overview of child physical abuse] 8 March 2015 ed. Göteborg: Internetmedicin.se, 2012.

87. Lindgren C. [Review of To tell the truth. Robert M Reece Titel: To tell the truth]. *Pediatrician* 2015(4):27.

88. Högberg U, Ludvigsson J, Thiblin I, Högberg G. [Shaken baby syndrome - pediatric, perinatal, forensic and psychiatric considerations] [Tuesday debate]. Stockholm: Swedish Society of Medicine, 2015.

89. Asplund K, Engström I, Wahlberg A, Pålsson Ahlgren C, Axberger HG. [Quick, incest and shaken baby syndrome – ethical issues regarding the role of experts in legal proceedings] Almedalen. Visby: The Swedish National Council on Medical Ethics (SMER), 2015.

90. Högberg G, Aspelin P, Högberg U, Colville-Ebeling B. Circularity bias in Abusive Head Trauma studies could be diminished with a new ranking scale. *Egyptian Journal of Forensic Sciences* 2016;6(1):6-10. doi: 10.1016/j.ejfs.2015.12.001 [published Online First: 14 January 2016]

91. Otterman G, Christian C, Dubowitz H, Frasier l, Glaser D, Greeley C, Harper N, Kelly P, Kemp A, Lucas L, Makoroff K, Palusci V, Pierce M-C "[International Course on Child Abuse Pediatrics, InterCAP2015, co-course director]. Uppsala: Section on Child Abuse and Neglect, American Academy of Pediatrics, the Ray E. Helfer Society, Swedish Paediatric Society, Uppsala University, 2015.

92. Wide P, Otterman O [Abusive Head Trauma: medical and legal perspectives] Annual meeting. Östersund: Swedish Paediatric Society, 2016.

93. Otterman G. [Paediatric abusive head trauma: experience in Sweden] The 36th Herbert Olivecrona symposium. Stockholm: Karolinska Intstitutet, 2016.

94. Otterman G. [Medical evaluation of suspected inflicted injuries in the young child] Annual Conference Stockholm: Swedish Society of Radiology, 2016.

95. SBU. Traumatic shaking – The role of the triad in medical investigations of suspected traumatic shaking: A systematic review. Stockholm: Swedish Agency for Health Technology Assessment and Assessment of Social Services (SBU), 2016:70.

96. Elinder G, Eriksson A, Hallberg B, Lynöe N, Sundgren P, Rosén M et al. Traumatic shaking: The role of the triad in medical investigations of suspected traumatic shaking. *Acta Paediatr* 2018;107 Suppl 472:3-23. doi: 10.1111/apa.14473 [published Online First: 28 August 2018]

97. Ludvigsson JF. [The SBU-report on shaking violence – far from clinical everyday life]. *Swedish Medical Journal* 2016;113(EDSA)

98. Otterman G. The sowing of unreasonable doubt: lessons learned and setbacks on paediatric abusive head trauma in Swedish courts. Paper Fifteenth International Conference on SBS/AHT. Montreal: National Center on Shaken Baby Syndrome, 2016.

99. Lucas S, Bertås A, Edstedt Bonamy AK, Törnudd L, Wide P, Otterman G. The way forward in addressing abusive head trauma in infants - current perspectives from Sweden. *Acta Paediatr* 2017 doi: 10.1111/apa.13840

100. Hellgren K, Hellstrom A, Hard AL, et al. The new Swedish report on Shaken Baby Syndrome is misleading. *Acta Paediatr* 2017 doi: 10.1111/apa.13845

101. Ludvigsson JF. Extensive shaken baby syndrome review provides a clear signal that more research is needed. *Acta Paediatr* 2017 doi: 10.1111/apa.13765

102. Lynöe N, Elinder G, Hallberg B, Rosén, Sundgren P, Eriksson A. Authors' overarching reply to all the responses received to the systematic literature review on shaken baby syndrome. *Acta Paediatr* 2017 doi: 10.1111/apa.13887

103. Lynöe N, Elinder G, Hallberg B, Rosén, Sundgren P, Eriksson A. Conflicts of interest issues. Response to Lucas et al. *Acta Paediatr* 2017 doi: 10.1111/apa.13891

104. Lynöe N, Elinder G, Hallberg B, et al. What are acceptable conclusions? Response to Dr Ludvigsson. *Acta Paediatr* 2017 doi: 10.1111/apa.13890

105. Bertås A. [Shaken baby syndrome – an issue that concerns us all] *Pediatrician* 2017(1):2.

106. Bondjers L, Wiksell Å, Räntfors J. [Infant abuse, investigation in case of suspicion of Abusive Head Trauma. Workmodel based on proposals for guidelines advocated by the the sub-association for Child Protection]. Annual meeting Göteborg: Swedish Paediatric Society

2017.

107. Bertås A. [Infant abuse, investigation in case of suspicion of Abusive Head Trauma. The SBU report – how everyday clinical practice is affected]

Annual meeting Göteborg: Swedish Paediatric Society, 2017.

108. Sennerstam R. [Shaken baby syndrome under scrutiny: a new orientation of the entire procedure is required]. *Pediatrician* 2017(1):28-29.

109. Otterman G. Unreasonable doubt: how Swedish courts and agencies are undermining the science in abusive head trauma cases. Annual Conference of the Ray E Helfer Society. Denver: Ray E. Helfer Society, 2017.

110. Andersson J, Thiblin I. National study shows that abusive head trauma mortality in Sweden was at least 10 times lower than in other Western countries. *Acta paediatrica* 2017 doi: 10.1111/apa.14138 [published Online First: Nov 6 2017]

111. [Court decision case 991-17, 9 February 2018]. Stockholm Swedish Supreme Administrative Court 2018.

112. Lynöe N, Elinder G, Hallberg B, Rosen M, Sundgren S, Eriksson A. [The conclusions of the SBU report about diagnostics of shaking baby syndrome remain] *Pediatrician* 2018(5):33.

113. Bertås A, Räntfors J, Wide P, Lucas S. [Rejoinder by Swedish Pediatric Society] *Pediatrician* 2018(5):33.

114. Räntfors J. [Collaboration and investigation of infant abuse] In: Society SP, ed. Annual meeting. Örebro: Swedish Paediatric Society, 2018.

115. Trysell K. [Pediatricians: Uncertainty has spread after the SBU report]. *Swedish Medical Journal* 2018;115:FCXM.

116. Wiksell Å, Caisander H. [Guidelines for radiology during investigation of suspected child abuse] Swedish Society for Pediatric Radiology, 2018.

117. Otterman G. Health sector and community response to child maltreatment in Sweden and in a European context. Uppsala University, 2018.

118. Jernbro C, Otterman G, Lucas S, Tindberg Y, Janson S. Disclosure of Child Physical Abuse and Perceived Adult Support among Swedish Adolescents. *Child Abuse Review* 2017 doi: 10.10002/car

119. Otterman G, Lainpelto K, Lindblad F. Factors influencing the prosecution of child physical abuse cases in a Swedish metropolitan area. *Acta Paediatr* 2013;102(12):1199-203. doi: 10.1111/apa.12399

120. Otterman G, Jalsenius M, Maguire S, Sarkadi A, Janson S. Paediatric approaches to child maltreatment are subject to wide organisational variations across Europe. *Acta Paediatr* 2017;106(7):1110-17. doi: 10.1111/apa.13779

121. Otterman G, Lahne K, Arkema EV, Lucas S, Janson S, Hellström-Westas L. Childhood death rates declined in Sweden from 2000 to 2014 but deaths from external causes were not always investigated. *Acta Paediatr* 2019;108(1):160-68. doi: 10.1111/apa.14309 [published Online First: 10 March 2018]

122. Högberg U, Lampa E, Högberg G, Aspelin P, Serenius F, Thiblin I. Infant abuse diagnosis associated with abuse head trauma criteria: incidence increase due to overdiagnosis? *Eur J Publ Health* 2018;28(4):641-46. doi: 10.1093/europub/cky062 [published Online First: April 17, 2018]

123. Högberg U, Andersson J, Squier W, Högberg G, Fellman V, Thiblin et al. Epidemiology of subdural haemorrhage during infancy: A population-based register study. *PLoS One* 2018;13(10):e0206340. doi: 10.1371/journal.pone.0206340 [published Online First: 2018/11/01]

124. Högberg U, Andersson J, Högberg G, Thiblin I Metabolic bone disease risk strongly contributing to long bone and rib fractures during early infancy: A population register study *PLoS ONE* 2018;13(12):e0208033. [published Online First: December 19, 2018]

125. Otterman O, Janson S. [Inflicted head injury of the infant]. In: Jägervall M, Lundgren J, ed. [Child neurology]

Lund: Studentlitteratur 2017.

126. Wide P, Laurell L. [Children who are abused]. In: Norgren S. LJ, Norman, M., ed. [Emergency Paediatrics]. Stockholm: Liber AB 2019:161-72.

127. Güvencel A. With what certainty can it be claimed that rib fractures or classical metaphyseal lesions in infants are attributed to physical abuse? A systematic literature review [MSc]. University of Dundee, 2019.

128. Protocol 27 March 2019 Stockholm: Council of National Board of Forensic Medicine 2019.

129. Otterman G. Workshop: Confronting Child Abuse Challenges in the Wake of the Swedish Agency for Health Technology Assessment (SBU) Report on Abusive Head Trauma (AHT). Ray Helfer Society Annual Meeting. Orlando: Ray Helfer Society, 2019.

130. Högberg U, Sennerstam R, Högberg G, Andersson J, Wester K, Thiblin I. Medical diagnoses among infants at entry in out-of-home care: A Swedish population-register study. *Health Science Report* 2019;2(8):e133. doi: 0.1002/hsr2.133 [published Online First: Jul 18]

131. Högberg U, Winbo J, Fellman V. Population-based register study of children born in Sweden from 1997 to 2014 showed an increase in rickets during infancy. *Acta Paediatr* 2019 doi: 10.1111/apa.14835 [published Online First: 2019/05/06]

132. Otterman G. The SBU Controversy – Is It Really? Seventh International Conference on Pediatric Abusive Head Trauma. Hershey: PennState College of Medicine, 2019.

133. Otterman G. A Flawed Report out of Sweden: Can the Institution Undermine Physicians and their Work? . Sixteenth International Conference on Shaken Baby Syndrome/Abusive Head Trauma. Orlando: National Center on Shaken Baby Syndrome 2019.

134. van Agthoven G. [Medical routine, management of suspected infant abuse]. Guideline Swedish Paediatric Society. Stockholm, 2019:9.

135. Hellgren K, Löfgren S, Lidén U, Austeng D, Teär Fahnehjelm K. [Retinal haemorrhage in violence to the head in young children. Proposal for clinical management support] *Swedish Medical Journal* 2019;117:19239.

136. Lynoe N, Eriksson A. [We look forward to the authors presenting their own fundus images] *Swedish Medical Journal* 2019;117:19239.

137. Thiblin I. [Haemorrhage due to vitreoretinal traction - truth or hypothesis]. *Swedish Medical Journal* 2019;117:19239.

138. Hellgren K, Löfgren S, Lidén U, Austeng D, Teär Fahnehjelm K. [Concluding remarks regarding AHT: Replies show the need for ophthalmologists competence in AHT] *Swedish Medical Journal* 2019;117:19239.
